# Advances and Challenges of Fluorescent Nanomaterials for Synthesis and Biomedical Applications

**DOI:** 10.1186/s11671-021-03613-z

**Published:** 2021-11-27

**Authors:** Deli Xiao, Haixiang Qi, Yan Teng, Dramou Pierre, Perpetua Takunda Kutoka, Dong Liu

**Affiliations:** 1 Department of Analytical Chemistry, China Pharmaceutical University, Nanjing, 210009 China; 2 Key Laboratory of Biomedical Functional Materials, China Pharmaceutical University, Nanjing, 210009 China; 3 Anhui Engineering Laboratory for Conservation and Sustainable Utilization of Traditional Chinese Medicine Resources, School of Biological and Pharmaceutical Engineering, West Anhui University, West of Yunlu Bridge, Moon Island, Lu’an, 237012 Anhui China

**Keywords:** Fluorescent nanomaterials, Carbon dots, Quantum dots, Silica nanoparticles, Metal nanoparticles, Biomedical applications

## Abstract

With the rapid development of nanotechnology, new types of fluorescent nanomaterials (FNMs) have been springing up in the past two decades. The nanometer scale endows FNMs with unique optical properties which play a critical role in their applications in bioimaging and fluorescence-dependent detections. However, since low selectivity as well as low photoluminescence efficiency of fluorescent nanomaterials hinders their applications in imaging and detection to some extent, scientists are still in search of synthesizing new FNMs with better properties. In this review, a variety of fluorescent nanoparticles are summarized including semiconductor quantum dots, carbon dots, carbon nanoparticles, carbon nanotubes, graphene-based nanomaterials, noble metal nanoparticles, silica nanoparticles, phosphors and organic frameworks. We highlight the recent advances of the latest developments in the synthesis of FNMs and their applications in the biomedical field in recent years. Furthermore, the main theories, methods, and limitations of the synthesis and applications of FNMs have been reviewed and discussed. In addition, challenges in synthesis and biomedical applications are systematically summarized as well. The future directions and perspectives of FNMs in clinical applications are also presented.

## Introduction

Conventional organic dyes have been facing some difficulties in their application in biomedicine due to their inherent defects like cytotoxicity and poor biocompatibility [1]. However, the emergence of fluorescent nanomaterials shows great potential in the replacement for conventional organic dyes. Scientists have poured much time and effort in the research of fluorescent nanomaterials, and the relevant achievements on synthesis and applications are more than inspiring.

The shape, size and structure of fluorescent nanomaterials determine their physical and chemical properties, which have huge influences on their performances. Hence, the controllable synthesis of fluorescent nanomaterials has become a hot research topic. The optimal experimental conditions of synthesis contribute to the most suitable size, morphology and stability of fluorescent nanomaterials. In recent years, much effort has been made to improve the biocompatibility of fluorescent nanomaterials by improving synthesis methods [2]. Metal ions were usually doped with carbon dots (CDs) or quantum dots (QDs) to functionalize the surface of fluorescent nanomaterials in the past. However, ineffective fluorescence and underlying toxicity posed a threat to their applications in bioimaging and biolabeling [3]. Considering these problems, Zuo et al. reported a high-efficiency CDs gene delivery system. CDs doped with fluorine were synthesized by solvothermal process, and positive charge sites for gene delivery can be provided by branched polyethyleneimine (b-PEI) [4]. It can be anticipated that new surface modification methods will be a hotspot research area in future.

**Fig. 1:**
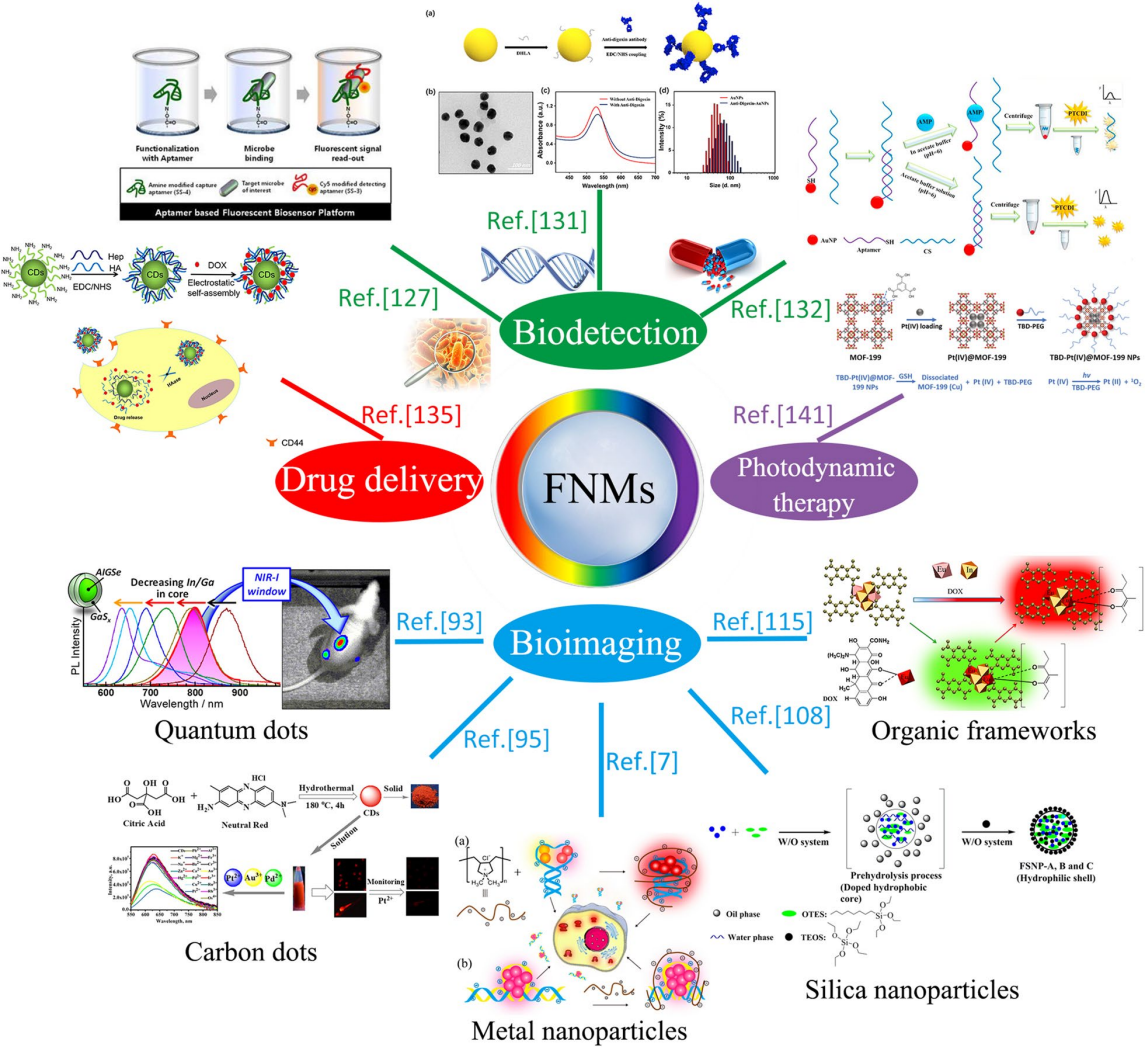
The overview diagram of biomedical applications of fluorescent nanomaterials

Many efforts have been made to explore the potential of fluorescent nanomaterials for biomedical applications which include bioimaging, biodetection and some therapy methods, as shown in Fig. 1. Reliable fluorescence for application depends on their physical and chemical properties [5]. Therefore, research work on improving their properties such as toxicity, hydrophilicity and biocompatibility has been a significant part of realizing the extensive use of fluorescent nanomaterials in biomedical areas. With the increasing rate of some diseases like cancer, there is an increasing demand for novel diagnosis and therapy strategies with higher accuracy and compliance from patients [6]. Currently, metal or non-metal ion doping and surface modification of fluorescent nanomaterials are still the dominant techniques in improving their PL efficiency and biocompatibility [7], and corresponding researches open up new visions of the biomedical applications of fluorescent nanomaterials.

Considering the great potential that fluorescent nanomaterials possess in the biomedical area, this review puts an emphasis on the latest advances and improvements. Scientists have devoted themselves to the functionalization of the surface of fluorescent nanomaterials and their performances in biomedical applications. Only with reasonable designed synthesis strategies can fluorescent materials be endowed with the quality of high PL efficiency and good biocompatibility, which are vital to their applications in the biomedical field. This review on the synthesis and applications of fluorescent materials, we hope, can be of some assistance to readers in understanding the general development trend of fluorescent nanomaterials currently.

## Synthesis of Fluorescent Nanomaterials

### Quantum Dots (Semiconductor Crystals)

Quantum dots (QDs) were the research spot in the past decades owing to their broad absorption and symmetric photoluminescence spectra, high quantum yield, high resistance to photobleaching, high molar extinction coefficients, and large effective Stokes shifts [8]. In terms of the formation mechanism of QDs, when charge carriers (electrons and holes) are restricted by potential barriers to certain regions, semiconductors show dramatic quantum size effects resulting in the shift of the absorption spectrum and fluorescence spectrum. The small regions are less than the de Broglie wavelength of the charge carriers, or equivalently, the nanocrystal diameter is less than twice the Bohr radius of excitons in the bulk material [9]. When the charge carriers are confined by potential barriers in three spatial dimensions, QDs are formed, which mainly consist of atoms from groups II-VI (CdSe, ZnS), III-V (GaAs, InP), or IV-VI (PbS, PbSe).

The synthesis of QDs was firstly reported in 1982 [10, 11]. Nanocrystals and microcrystals of semiconductors were grown in glass matrices. With the development of fluorescent materials, QDs have been prepared by different methods, such as direct adsorption method, linker-assisted adsorption method, situ methods and the combination of previous preparation methods. The combination of previous methods includes combination of prepared semiconductors with QD precursors and combination of previously prepared QDs with semiconductor precursors, in which the semiconductors or QDs are prepared separately [12].

After a series of researches on the synthesis of QDs, plenty of researchers reported the fluorescent property study of QDs. Bawendi et al. synthesized the QDs with narrow size distributions via introducing semiconductor precursors such as cadmium sulfide (CdS), cadmium selenide (CdSe), or cadmium telluride (CdTe) to investigate the size-dependent optical properties of QDs [13]. Since then, CdSe became the most common chemical composition of QDs, and a variety of surface modifications [14–16] or protective inorganic shell [13, 17] have been utilized to render colloidal stability.

### Carbon Dots

Carbon dots (CDs) are emerging nanomaterials in the nanocarbon family with sizes less than 10 nm, which were firstly obtained in the purification of single-walled carbon nanotubes (SWCNTs) by electrophoresis in 2004 [18]. It is noteworthy that CDs gradually substitute semiconductor quantum dots on the grounds of high solubility in water, low cytotoxicity, high photostability, excitation-dependent multicolor emission, preferable flexibility in surface modification, excellent cell permeability, and better biocompatibility [19, 20]. Generally, the CDs mainly comprise carbon quantum dots (CQDs) and graphene quantum dots (GQDs). A mass of synthetic methods for CDs with tunable sizes can be broadly divided into two main groups: chemical methods and physical methods [21].

#### Chemical Synthetic Methods

Chemical synthetic methods are the most commonly used in the preparation of carbon dots because the resultant CDs possess excellent properties, such as superior water solubility, chemical inertness, low toxicity, ease of functionalization and resistance to photobleaching. In general, chemical synthetic methods include electrochemical synthesis [22, 23], acidic oxidation [24, 25], hydrothermal carbonization [26], microwave-assisted/ultrasonic treatment [27–29], solution chemistry methods [30], supported synthesis [31], etc.

Among the numerous synthetic methods, electrochemical synthesis has been repeatedly reported over the past decades. Zhao’s group reported a novel method of preparing CDs with low cytotoxicity by electrooxidation synthesis, whereby the CDs were prepared via oxidizing a graphitic column electrode against a saturated calomel electrode with a Pt wire counter electrode in NaH_2_PO_4_ aqueous solution [22]. The supernatant was then ultrafiltered through centrifugal filter devices to obtain the CDs with blue and yellow fluorescence, respectively. Another direct electrochemical approach was recently reported by Qu et al., for the GQDs with a uniform size of 3-5 nm by electrochemical oxidation of a graphene electrode in phosphate buffer solution [23]. The photoluminescent (PL) color of these particles was green.

Mao et al. completed the combustion oxidation synthesis of CDs in 2007 by mixing candle soot with an oxidant, followed by refluxing, centrifugation and dialysis to purify the CDs. The prepared CDs photoluminescence spectra have a broad color range, with the emission-peak wavelengths ranging from 415 (violet) to 615 nm (orange-red). Then, the obtained CDs were further subjected to polyacrylamide gel electrophoresis to separate the CDs with different optical characterization. Acidic oxidation has also been extensively used for the preparation of stable nanomaterials such as carbon dots. After the acid treatment of carbon nanotubes/graphite and refluxing, the resultant CDs of 3-4 nm presented a tan transparent liquid which emitted bright yellow fluorescence under ultraviolet light and were quite stable in saline. This made CDs with longwave (yellow/orange/red) fluorescence possess better penetration. The CD solution can be preserved at room temperature for a long time and no precipitates are formed that cause the loss of fluorescence [25].

Microwave/ultrasonic synthesis has gradually and mainly become an auxiliary synthetic technology in the process of synthesis [32]. Fluorescent CDs, 3–5 nm in diameter, were synthesized by Xiao’s group via an economical, rapid and green microwave-assisted approach [33]. The most salient feature of this one-step approach was that both the formation and functionalization of CDs were completed simultaneously through the microwave pyrolysis derived from the ionic liquids for the first time [34]. The reaction process occurred in a microwave oven using cheap ionic liquids as the source of carbon and the solution changed from colorless to dark brown as the reaction time went on [35]. Tang et al. used an ultrasonic method on the basis of glucose or active carbon as the source of carbon to synthesize monodispersed water-soluble CDs. They emitted bright and colorful fluorescence [28]. Similarly, Vanesa Romero et al. obtained highly fluorescent nitrogen (N) and sulfur (S) co-doped carbon dots (CDs) after photochemical oxidation of the carbohydrates in vegetables. The co-doping of N and S increases the number of active sites on the CDs surface, thus enhancing its luminescence performance [36]. Nitrogen-doped carbon quantum dots (NCQDs), a fluorescent probe, were successfully applied to the determination of doxycycline [37]. Pathak et al. prepared co-doped carbon dots with nitrogen and sulfur (NSCDs) as well, which were synthesized from thiourea and tris–acetate-ethylenediamine buffer by microwave hydrothermal method. The NSCDs were put into use to image various pathogenic bacteria and human buccal epithelial cells due to multicolor fluorometry [38].

Considering that most of the above mentioned synthetic methods needed strong acid, several complicated experimental steps, and further modifications with other compounds to improve the water solubility of the CDs and enhance their photoluminescence property, some research teams exploited the hydrothermal carbonization of carbohydrates photoluminescence such as chitosan, glucose, citric acid, etc. to avoid the complex and time-consuming purification and functionalization processes [39]. Yang et al. described a one-step synthetic method for highly amino-functionalized fluorescent CDs with a quantum yield (QY) of 7.8% by hydrothermal carbonization of chitosan at a mild temperature. This method needed neither a strong acid solvent nor surface passivation reagent. Besides, the functional groups on the CDs’ surface improved their water solubility and reduced their potential biotoxicity [26]. The multi-doped carbon dots (MCDs), with bright and color-tunable emission, were synthesized by one-pot method without any further surface passivation. The synthesized MCDs were doped with abundant biogenic elements (O, N, P) and therefore display strong fluorescent emission and excitation-wavelength-dependent characteristic, good aqueous solubility, high optical stability as well as ion-stability. The MCDs not only can selectively and sensitively detect Fe^3+^ under blue light of detection at 15.9 nm, but also measure the intracellular Fe^3+^ through multi-color fluorescence imaging [40].

For solution chemistry methods, oxidative condensation of aryl groups has been successfully applied to the preparation of GQDs over the past decades. Stable colloidal GQDs with desired sizes and structures were produced by Li’s group with solubilization strategy. This method achieved size tunability and narrow size distribution of CDs without any impractical size separation process [30]. When it comes to the supported synthetic procedure, a number of research teams have taken advantage of it to complete the synthesis of monodisperse nanomaterials such as nanosized CDs. Zhu’s group adopted mesoporous silica (MS) spheres as nanoreactors and citric acid as the carbon precursor and hydrophilic CDs with the sizes of 1.5–2.5 nm were prepared by an impregnation method. The CDs with a high photoluminescence efficiency of 23% were capable of emitting strong blue luminescence and presenting excellent conversion luminescence properties [31]. Bright-yellow-emissive carbon dots (Y-CDs) were prepared by Yan et al. through solvothermal method, using anhydrous citric acid as carbon source and 2, 3-phenazinediamine as nitrogen source. The Y-CDs with abundant carboxyl groups displayed a respectable fluorescence quantum yield (24%), 188-nm Stokes’ shift, high sensitivity and excellent stability [41]. The synthetic methods and properties of CDs are presented in Table 1.

**Table 1:** Chemical synthetic methods and properties of carbon dots

Methods	Doped elements	Size	PL color	QY (%)	Ref
Electrochemical synthesis	-	1.5–4 nm	Blue/yellow	1.2 (blue CNCs)	[22]
Electrochemical synthesis	-	3–5 nm	Green	-	[23]
Combustion/acidic oxidation	-	< 2 nm	Violet/red/yellow	< 2	[24]
Combustion/acidic oxidation	-	3–4 nm	Yellow	~ 3–6	[25]
Microwave/ultrasonic treatment	-	3–5 nm	Blue	5.18	[33]
Microwave/ultrasonic treatment	N, S	2.92 nm-4.48 nm		57	[38]
Microwave/ultrasonic treatment	-	1.65 nm	-	7–11	[28]
Microwave/ultrasonic treatment	N, S	8 nm	Blue	22	[36]
Hydrothermal carbonization	O, N, P	1–6 nm	Bright and color-tunable	14.5	[40]
Hydrothermal carbonization	-	4–7 nm	Blue	7.8	[26]
Hydrothermal carbonization	N	2.70 nm	Blue	23.48	[42]
Microwave-hydrothermal carbonization	N, O	-	Blue	45.9	[43]
Solution chemistry methods	-	Tunable	-	-	[30]
Supported synthesis	-	1.5–2.5 nm	Blue	23	[31]
Solution chemistry methods	-	7.2 nm	Yellow	24	[41]

#### Physical Synthetic Methods

In general, physical synthetic methods mainly include arc discharge, laser ablation/passivation, and plasma treatment. Xu and co-workers oxidized the arc-discharge soot with HNO_3_ and then separated the suspension by gel electrophoresis into SWCNTs. They finally isolated the fast moving band of highly fluorescent carbon dots nanoparticles [18]. CDs using nano-carbon materials as the precursor and an environment-friendly solvent as the liquid medium were prepared by Li et al. via a mild laser ablation approach [44]. In addition, Gokus and co-workers demonstrated that using oxygen plasma could induce the strong fluorescence into single-layer graphene [45].

### Carbon Nanoparticles

Fluorescent carbon nanoparticles, with their reduced cytotoxicity, resistance to photobleaching, and increased biocompatibility, are attracting increasing attention for bioimaging and other biomedical applications. Compared with the typical sizes of carbon dots within 1–6 nm, the sizes of carbon nanoparticles are more than 20 nm, which saves the trouble to separate, purify and collect [46]. Synthesis methods for carbon nanoparticles are similar to carbon dots, including hydrothermal carbonization, microwave treatment, chemical ablation method and laser ablation. These methods have their own advantages but cannot effectively control the size of nanoparticles. Electrochemical carbonization is a single step method which can control the size and luminescence properties of carbon nanoparticles. Unfortunately, there are only very few substrates available for this method. At present, some new intriguing methods have been reported such as phosphorus pentoxide combustion method [47].

In recent years, carbon nanoparticles suitable for biomedical applications are synthesized with modified methods. Santu et al. resolved the synthesis of high quality red fluorescent carbon nanoparticles by controlled carbonization of resorcinol [48]. This approach involves oxidative phenol coupling associated with dehydration to form red fluorescent carbon nanoparticles. Anara et al. synthesized fluorescent carbon nanoparticles with quantum yield of 6.08% using a modified hydrothermal method. Compared with the conventional methods which require long thermal treatment up to several hours, this method shortened the reaction time to less than 30 min, realizing the rapid synthesis of fluorescent carbon nanoparticles [46].

### Carbon Nanotubes

One-dimensional (1D) carbon nanotubes have generated enormous attention in biomedical field by virtue of their excellent electronic and optical properties. Carbon nanotubes can be divided into single-walled carbon nanotubes (SWCNTs) and multi-walled carbon nanotubes (MWCNTs) according to the number of cylindrical graphene layers. While SWCNTs are composed of a single layer of graphene sheet rolled into a cylinder, MWCNTs comprise several concentric layers of graphene sheet. The outer diameter of carbon nanotubes is below 100 nm, but their lengths can reach as long as several millimeters, which leads to very high aspect ratio and large surface area [49]. Moreover, the unique arrangement of carbon atoms in carbon nanotubes forms a rich π-electron conjugation outside the nanotube [50]. In addition, carbon nanotubes are endowed with strong absorption and fluorescence in NIR region [51]. All of these characteristics contribute to effective interaction with biomolecules, which makes carbon nanotubes an ideal candidate for biomedical applications.

Synthetic methods have great influence on the diameter, length, structure, chirality and quality of carbon nanotubes, and in the meanwhile, it should be considered whether this method is amenable to large-scale production. Commonly used methods include arc-discharge [52], laser ablation [53] and chemical vapor deposition [54]. On top of this, carbon nanotubes need to be functionalized to improve their solubilty and prevent them from aggregating in solvents and biological media. Covalent functionalization would introduce defects to the structure of carbon nanotubes, leading to dramatical decrease or even complete loss of their NIR fluorescence. Noncovalent functionalization with amphiphilic molecules such as polymers would preserve the structure and fluorescent properties of carbon nanotubes, but lower the QY of carbon nanotubes. In order to overcome these obstacles, novel methods to synthesize and functionalize carbon nanotubes have been reported recently. Lee et al. reported that the addition of dithiothreitol, which is a reducing agent, can enhance the fluorescent QY of SWCNTs for the first time, resulting in fluorophores having brightness equivalent to that of QDs [55]. Hou et al. investigated the addition of dithiothreitol to SWCNTs functionalized with a variety of surfactant. For DNA and SDS wrapped SWCNTs, the fluorescent QY of them increased significantly, while fluorescence quenching to different extents was observed for other surfactant [56]. As a result, the addition of dithiothreitol to DNA or SDS wrapped SWCNTs are feasible solutions to achieve the application of carbon nanotubes in biomedicine.

### Graphene-Based Nanomaterials

As two-dimentional carbon nanomaterials, graphene and its derivatives have been widely explored for a range of biomedical applications such as bioimgaing and drug delivery. Graphene nanomaterials include graphene nanosheet, graphene oxide (GO) and reduced graphene oxide (rGO) nanosheet. They have high surface areas and unique surface properties which allow noncovalent interactions with dye molecules, biomolecules and water-insoluble drugs. Many researchers have reported different graphene preparation methods since it was prepared successfully for the first time in 2004. Synthetical methods of graphene nanomaterials can be classified into two categories, the top-down and the bottom-up.

Top-down methods involve the isolation from stacked graphite layers to form graphene sheets, including mechanical exfoliation [57], solvent-based exfoliation [58] and electrochemical exfoliation [59]. Gu et al. systematically studied ultrasound-assisted solvent based exfoliation, and found that ultrasonic waves have a good exfoliation effect. They can also affect the size and thickness distribution of graphene sheets, which makes controllable synthesis possible. Bottom-up approaches involve reorganization of carbon atoms using alternative carbon sources. Epitaxial growth [60] and chemical vapor deposition (CVD) [61] are the most commomly used bottom-up synthesis methods. GO sheets composed of many sp^2^ domains isolated by oxygen-containing groups can be synthesized using Hummer’s method. Variations in sizes of these sp^2^ domains make the PL of GO sheets widely range from 500 to 800 nm [62]. rGO is derived from GO through chemical reduction using reducing agents such as hydroquinone and hydrazine. Compared with GO, the fluorescence of rGO showed blue-shifted emission in the UV region along with fluorescent quenching, which is attributed to the percolation pathways between the newly formed crystalline sp^2^ clusters [63]. Akbari et al. elucidated that the ratio of sp^3^/sp^2^ domains in GO sheets determines their fluorescence spectra. Therefore, GO is a promising fluorescent nanomaterial over a wide range of wavelengths under different degrees of reduction, which can be used in the biomedical applications.

### Metal Nanomaterials

Noble metal atoms present less cytotoxicity compared with QDs at the same time. Gold, silver and copper nanoparticles have received increasing attention and are applied to a large number of fields. In the biomedical fields, the quantum mechanical effects of gold nanoparticles, such as photoluminescence emission or plasmon resonance make gold nanoparticles (AuNPs) an ideal candidate for another in vivo nanosensor with low cytotoxicity [64, 65].

AuNPs have attracted extensive scientific interest by virtue of their ease of synthesis and unique properties, and diverse synthetic methods have been reported. As one of the most important methods, chemical methods are generally performed by treating an aqueous solution of chloroaurate with reducing agents in the presence of a stabilizing agent. Citric acid is mostly widely used, which can act as both a stabilizer and reducing agent [66]. However, AuNPs stabilized with citric acid can go through irreversible accumulation during functionalization development with thiolate ligands. This problem can be overcomed by making the reaction take place in the presence of water-soluble polymers, surfactants, or capping agents that help to provide higher stability and prevent nanoparticles aggregation. The size and shape of AuNPs can be controlled by changing the gold-citrate proportion, surface-modifying agents or reaction conditons. With one-pot ultrasonic emulsification method, Zhang and his coworkers co-loaded Bis(4-(N-(2-naphthyl) phenylamino) phenyl)-fumaronitrile and AuNPs into micelles to obtain the nanoprobe [67]. Most of all, the obtained nanoprobe, with great potential to be applied in tumor-targeted imaging and diagnosis in vivo, processed excellent fluorescence imaging capacity, despite the existence of gold nanoparticles. Although AuNPs are non-toxic under certain experimental conditions, the toxicity and the side effects need to be thoroughly examined [68].

Fluorescent Ag nanoclusters have been paid much attention due to their unique physical and chemical properties. The synthesis process of such nanoclusters is classified by the stabilizing scaffold into DNA oligonucleotides, peptides, proteins, dendrimers and polymers. Besides, extensive literature has demonstrated some green synthesis, such as the application of aqueous stem extracts of *D. trifoliata and S. alba* to optimize the preparation conditions [69].

Cu nanoclusters (Cu NCs) are relatively widely-used as noble metal materials, but their synthesis is still scarce due to their vulnerability to oxidation. Recently, Kawasaki et al. successfully prepared stable Cu NCs by a microwave-assisted polyol method [70]. DNA could be used as the template for the synthesis of fluorescent Cu NCs. Mohir et al. proposed a method based on double-strand DNA in solution to obtain Cu NCs with high selectivity [71]. Using fluorescent properties of Cu NCs, it was successfully exploited as an effective fluorescent turn-on signal indicator for the selective determination of RDX [72].

### Silica Nanoparticles

Considering the properties of transparency, mechanical stability, robustness and stabilization of the embedded fluorophores, silica nanoparticles are extensively applied in biological domains. For example, silica core/shell NPs applied for the intracellular detection of Zn^2+^ and H_2_PO_4_^−^ in living cells were synthesized as “off/on” fluorescent nanosensors. In recent years, silica nanoparticles doped with organic dyes have been synthesized and widely used in many applications such as biodetection [73]. The most widely used synthesis methods for silica nanoparticles are the Stöber method and the reverse microemulsion method. The Stöber method, first described in the 1960s [74], involves the hydrolysis of alkyl silicates and subsequent condensation of silicic acid in alcoholic solutions catalysed by the addition of ammonia. The second method of silica NP formation, the reverse microemulsion method, involves the reaction of alkyl silicates, typically TEOS, inside the water droplets of a water-in-oil microemulsion [75]. He et al. prepared three kinds dye doping silica nanoparticles with Stöber method and reverse microemulsion method by embedding dye into the core of the particle. Some molecules functionally responsive to Zn^2+^ are deposited on particle surfaces [76]. The fluorescent silica nanoparticles were used for fluorescent images intracellular Zn^2+^ (H_2_PO_4_^−^) in HeLa cells. When Zn^2+^ was added to the proportional Zn^2+^ nanosensor, the nanoparticles showed the ability to ratiometrically detect the concentration of H_2_PO_4_^−^.

In general, silica nanocomposites with good monodispersity and biocompatibility can be easily further modified with functional groups [77–79]. Lee et al. doped magnetic nanoparticles and fluorescent dyes into silica nanoparticles. These silica nanocomposites can be used not only as multimodal imaging probes for magnetic resonance (MR) and fluorescence imaging, but also as anticancer drug delivery carriers [80]. In a nutshell, silica particles could be a research spot in such cases with greatly extended uses.

### Phosphors

Phosphors are widely used in biomedicine owing to their unique advantages in reducing the autofluorescence and light-scattering interference from tissues. In general, phosphors are composed of host materials and doped ions [81]. Among the host materials of phosphors, yttrium oxide (Y_2_O_3_) is more than promising due to its low photodurability and its phonon energy. Lanthanides are largely doped in phosphors considering their abundant electron levels and energy transfer channels. Numerous methods have been reported for the preparation of phosphors including hydrothermal [82], flame spray pyrolysis [83], sol–gel [84], and co-precipitation processes [85].

Hydrothermal synthesis is emerging as an ideal process, which have been proven to be efficient and economical for the synthesizing of phosphors. Yu et al. synthesized Y_2_O_3_:Eu^3+^ phosphors by hydrothermal method in the presence of sodium citrate [82]. The concentrate of sodium citrate, the addition amount of NaOH and Eu in the hydrothermal process decided the properties of the obtained phosphors. Flame spray pyrolysis is a promising methods for the rapid and consecutive synthesis of oxide-based phosphors. Compared with conventional methods, this method provides phosphors with high crystallinity and homogenous dopant distribution. Khan et al. successfully produced Tb^3+^–doped Y_2_O_3_ phosphors that were approximately 100 nm in diameter with a narrow size distribution using flame spray pyrolysis [83]. In their method, alkali salt was mixed with other metal nitrate precursors, which effectively controlled size distribution in a narrow range. The sol–gel synthesis route offers several advantages, such as high homogeneity and purity, reduced synthesis time, uniform particle morphology, and narrow particle size distribution [86]. Leonardo et al. obtained Sm^3+^ doped SiO_2_-Gd_2_O_3_ phosphors by a sol–gel process [84]. Co-precipitation is a common and simple method for synthesizing crystalline phosphors, which ensures high homogeneity and controlled morphology characteristics. Perhaita et al. reported that the phase composition of the phosphors strongly depends on the pH during the precipitation [85].

### Organic Frameworks

Covalent-organic frameworks (COFs) are new porous crystalline materials possessing outstanding stability, adsorption and low toxicity. The design of fluorescent small organic molecules with a combination of fluorescence determination methods can be used to construct more efficient nanoprobes [87]. For selective 2,4,6-trinitrophenol (TNP) determination, a novel Naphthalimide-Benzothiazole conjugate was prepared as colorimetric and fluorescent nanoprobe. The fluorescence emission peaks of receptor were selectively quenched by TNP with a limit of detection as low as 1.613 × 10^–10^ M.

Metal organic frameworks (MOFs) are a kind of new generation multifunctional inorganic–organic materials with various holes and functionalized 3D crystalline structures formed by metal ions and linkers. MOFs show potential applications in separation, catalysis and other aspects due to unique attributes such as excellent chemical tenability, specific surface area and confinement of the pores. Some of the MOFs are luminescent and the quantum yield as well as light intensity will be influenced by temperature and excitation wavelength [88]. With the addition of doxycycline, Yu et al. synthesized a new functional metal–organic framework of pyromellitic acid and europium, which exhibited remarkable fluorescence enhancement at 526 nm and 617 nm. Results showed that both fluorescence intensities were positively correlated with the doxycycline concentration. The unique fluorescence response of the system could discriminate doxycycline from other tetracycline antibiotics with high selectivity.

## Biomedical Applications of Fluorescent Nanomaterials

### Bioimaging

#### Quantum Dots for Bioimaging

Fluorescent nanomaterials have been widely used in bioimaging. Compared with conventional organic fluorescent molecules, fluorescent nanomaterials are equipped with many superior properties such as high photostability, tunable emission spectra and high quantum yields [89].

As early as 1998, QDs were first successfully applied in biological imaging [90]. Since then, applications of QDs in this field have been springing up gradually. Chen’s group applied it in bioimaging and nuclear targeting with great stability and biocompatibility in living cells [91]. In spite of the extremely high sensitivity and spatial resolution of QDs, poor performances on hydrophilicity and biocompatibility hindered their applications in bioimaging in vivo. To tackle this problem, it has been found that the water-solubility of QDs can be greatly improved by attaching thiol or other hydrophilic groups to the surface of quantum dots [92]. In the same way, with the intention of improving the effectiveness and specificity of in vivo targeted imaging, targeting molecules are attached to the surface of QDs. Furthermore, the wavelength region of the emission light can be controlled by altering the size of QDs.

**Fig. 2:**
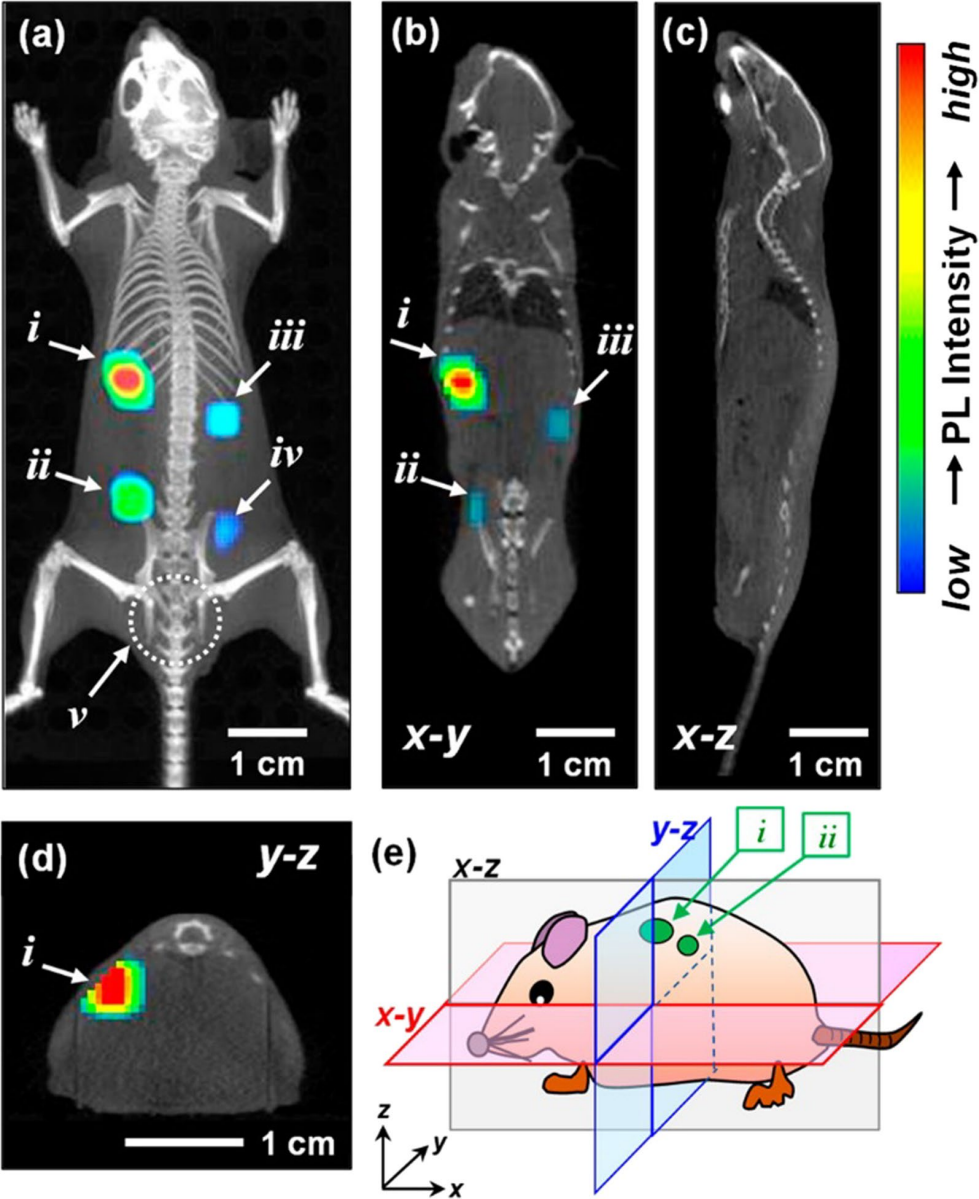
Three-dimensional PL image superimposed on an X-ray CT image of the mouse subcutaneously injected with DSPC-AIGSe@GaS_x_ liposome dispersions (each 50 mm^3^) in the back [78]

The combination of QDs and inorganic metal ions can optimize the application of QDs in bioimaging because the QDs’ defect-site PL peaks will be utterly removed by controlling the proportion of doped inorganic metal ions. Kuwabata, S et al. modulated the degree of Ga^3+^ doping in Ag–In–Se QDs. Thus, the QDs’ defect-site PL peaks were completely removed and a sharp band-edge emission peak come into appearance [93]. They found a blue shift of the band-edge PL peak ranging from 890 to 630 nm which could be credited to the fact that the energy gap of QDs was enlarged by Ga^3+^ doping. After injecting a mouse with QDs, the potential of AIGSe@GaS_x_ core–shell QDs for bioimaging turned out satisfying. The imaging effect of this kind of QDs in mice is demonstrated in Fig. 2. However, sensing of mid-IR wavelengths is challenging due to increased dark currents and noise. HgTe QDs synthesized by colloidal method is a promising candidate for IR bioimaging by virtue of lower dark currents, higher-temperature operation, and higher detectivity [94].

#### Carbon Dots for Bioimaging

**Fig. 3:**
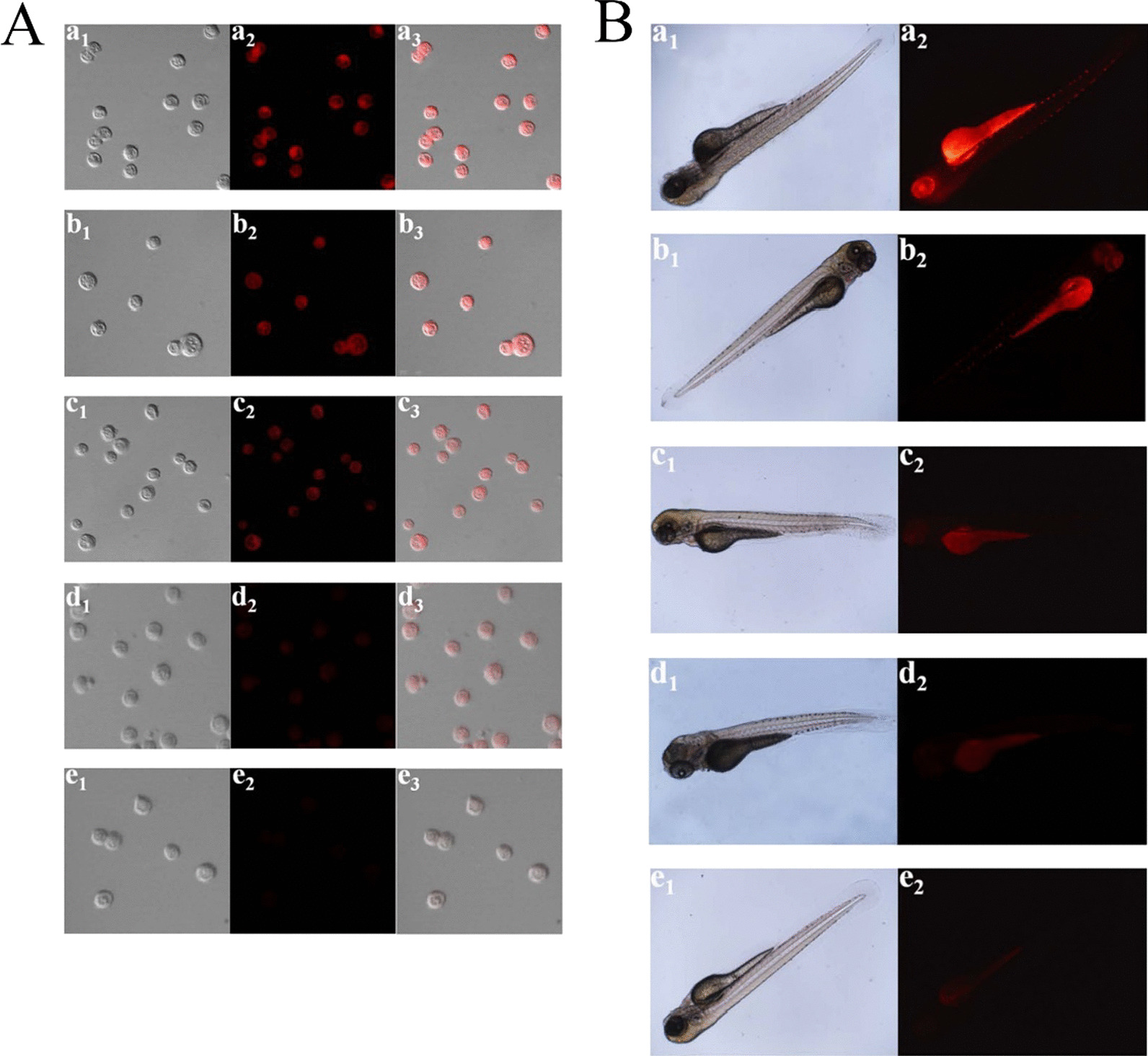
**A** Confocal imaging of Pt^2+^ in PC12 cells. (a1–e1) Bright field images. (a2–e2) Black field images of the CDs in PC12 cells with the different concentrations of Pt^2+^ (0, 25, 50, 150, and 300 μM). (a3–e3) Overlay images. **B** Fluorescence imaging of Pt^2+^ in ZF. (a1–e1) Bright field images. (a2–e2) Fluorescence images of the CDs in ZF with the various concentrations of Pt^2+^ (0, 30, 60, 100, and 150 μM) [80]

**Fig. 4:**
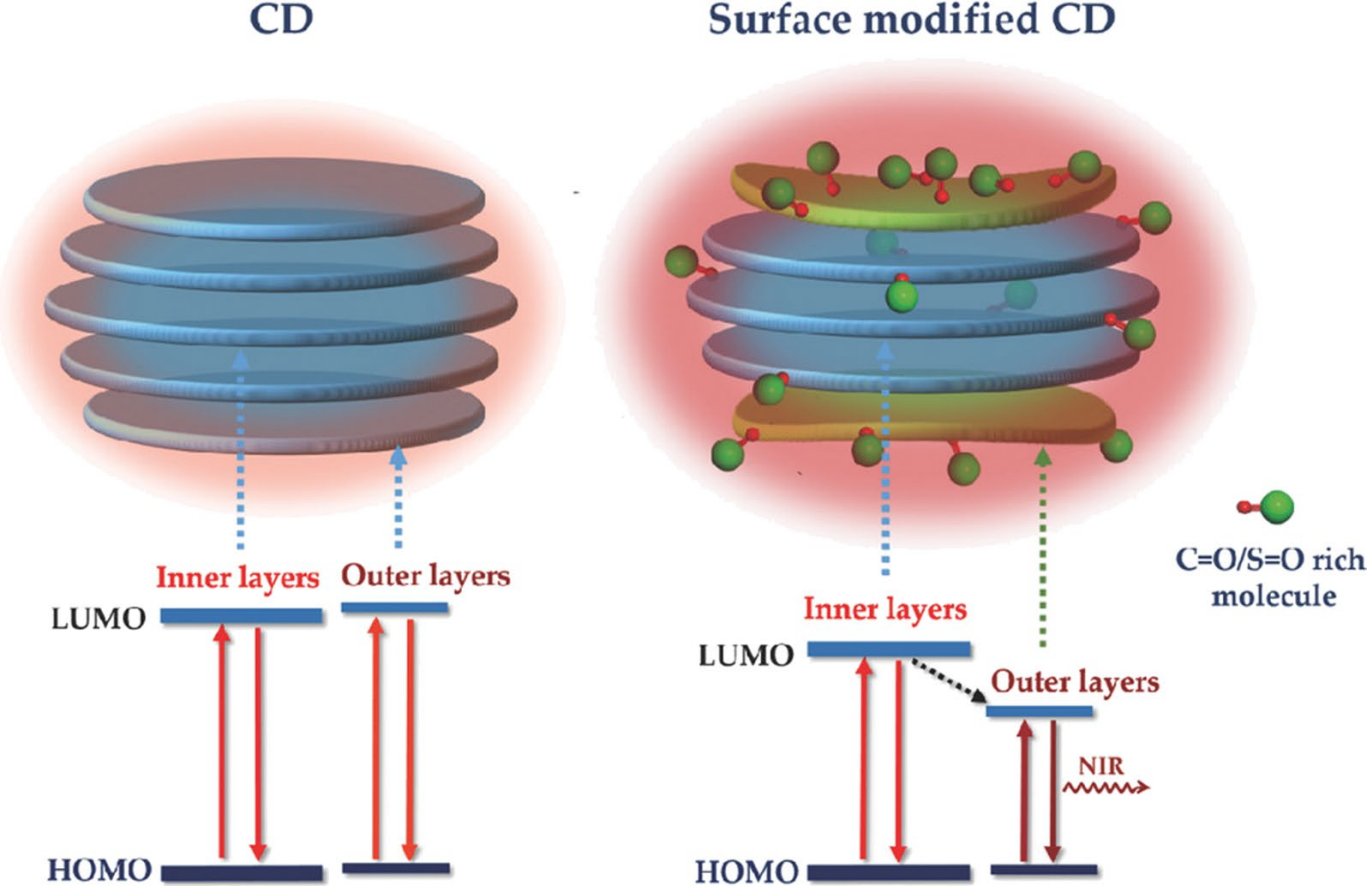
Schematic of structure and energy level alignments of nontreated CDs (left column) and CDs modified with S = O/C = O‐rich molecules (right column). The red (oxygen atom) and green double‐bonded balls represent the C = O/S = O‐rich molecule [81]

The poor photostability of current fluorescent nanomaterials hinders their long-term bioimaging to a large extent. To overcome this limitation, CDs have been studied for bioimaging and some positive results have been obtained due to the great performance on PL efficiency. Enormous efforts have been put to improve their water solubility and lower their toxicity in organisms. At present, most CDs are facing a barrier in bioimaging, that is, their short-wavelength excitation disables deep penetration in tissue. Aside from this, being exposed under the short-wavelength for a long time could do irreversible damages to living cells and tissues. As shown in Fig. 3, with the purpose of overcoming this deficiency, Gao et al. designed fluorescent CDs with red emission which were successfully used for bioimaging of noble metal ions (Pt^2+^, Au^3+^, Pd^2+^) in cells and zebrafish [95]. Sun and co-workers first studied the near infrared (NIR) imaging of CDs in vivo using mice as a model. Recently, it was reported that molecules or polymers containing plentiful sulfoxide or carbonyl groups can enhance NIR fluorescence through the surface modification. As shown in Fig. 4, under NIR excitation, sulfoxide or carbonyl groups are bound to the outer layers and the edges of the CDs. Thus, electron transitions are promoted, influencing the optical bandgap [96].

#### Carbon Nanoparticles for Bioimaging

In the field of bioimaging, fluorescent carbon nanoparticles show unique chemical and optical properties over traditional fluorescence probes. Different size, shape and elemental composition make carbon nanoparticles with different features. The biomedical fields are always seeking the most promising fluorescent carbon nanoparticles. Gaurav et al. obtained both larger and smaller size carbon nanoparticles with laser ablation method [97]. Both green and blue fluorescence were observed in the cells incubated with the carbon nanoparticles, suggesting their different sizes. Cell viability results indicated that the prepared carbon nanoparticles were nontoxic and safe for bioimaging applications. Shazid et al. employed carbonization method to obtain fluorescenct carbon nanoparticles derived from biocompatible hyaluronic acid. Both the in vitro and in vivo bioimaging studies showed that the prepared carbon nanoparticles would be reliable and stable for opticle imaging. Moreover, based on the experimenal data, their cytotoxicity was proved to be tolerable for biomedical applications.

#### Carbon Nanotubes for Bioimaging

Fluorescence of carbon nanotubes in the NIR is attracting high attention for their good light penetration depth in biological tissues. However, their low quantum yield requires for considerable excitation doses, leading to a fair degree of blue-shift and failure of penetrating live tissue. Mandal et al. reported that bright and biocompatible p-nitroaryl functionalized SWCNTs, encapsulated in phospholipid-polyethylene glycol, are suitable for bioimaging applications. The prepared SWCNTs enabled high signal-to-noise ratio imaging in live brain tissues using ultra-low excitation intensities. Their 1160 nm emissions in the NIR guarantee that they will provide optimal fluorescence imaging results [98]. Ceppi et al. applied SWCNT-based fluorescence imaging to debulking surgery in an ovarian cancer mouse model. SWCNTs are coupled to an M13 bacteriophage carrying modified peptide binding to the SPARC protein, which is overexpressed in ovarian cancer, leading to real-time imaging to guide intraoperative tumor debulking. This imaging system enables detection in the NIR window with a pixel-limited resolution of 200 μm, demonstrating real potential in fluorescence imaging guided surguries for patients [99].

Furthermore, fluorescent moieties can be conjugated by a carbon nanotube backbone, which integrate strong fluorescent ability with robust mechanism strength, exhibiting ideal bioimgaing results. Katharina et al. functionalized SWCNTs with an amphiphilic C_18_-alkylated polymer conjugated with bright perylene bisimide fluorophores. The polymers wrapping around the SWCNT backbones not only increase their water dispersibility but also promote their biocompatibility by providing a shield. In vitro studies on HeLa cells demonstrated that the biocompatibility of SWCNTs is dramatically improved. In microscopy studies, direct imaging of the SWCNTs' cellular uptake via perylene bisimide and SWCNT emission proved their potential for bioimging [100]. Park et al. combined carbon nanotubes with mussel adhesive proteins which can be specifically targeted at tumors in tissue. They then made carbon nanotubes conjugated with a ZW800 NIR fluorophore to obtain NIR fluorescence imaging [101]. The prepared carbon nanotube probes react with a specific tumor in one hour and can be easily eliminated via urine, demonstrating great value as tumor imaging and detecting agent.

#### Graphene-Based Nanomaterials for Bioimaging

Large surface area and feasible further functionalization make graphene-based nanomaterials a promising candidate for biomedical applications. However, as a result of their chemical sturctures, graphene nanosheets lack photoluminescence and rGO only display weak fluorescence, which makes it difficult to be utilized in bioimgaing applications. Many researchers attempted to resolve this problem by conjugation of fluorescent dyes and probes onto the large surface of graphene and its derivatives. Sun et al. reported an assembly strategy to prepare fluorescence probe RACD functionalized a single layer GO via π-π interaction and hydrogen bonding. The resluting nanomaterials exhibited that the fluorescent probes reduce the aggregation degree and acquire very well monodispersion, hydrophilicity and photostability, which is attributed to the strong synergy between RACD and GO [102]. Even so, fluorescence quenching remains a critical issue for these materials. In addition, the biocompatibility and toxicity of polymers applied to connect graphene-based materials and fluorescent moieties have not been adequately investigated. These facts appeal for alternative solutions to utilize graphene and its derivatives in bioimaging applications. Georgia et al. developed intrinsically photoluminescent graphene derivatives that show desirable biocompatibility and tunable fluorescence properties [103]. They can be organophilic or hydrophilic with different amine functionalization dodecylamine and hexamethylenediamine, respectively. The intrinsic fluorescent graphene-based nanomaterials possess great potential in a variety fileds of bioimgaing.

#### Metal Nanomaterials for Bioimaging

In recent years, fluorescent metal nanoparticles have shown great potential in bioimaging for improved disease diagnosis and treatment [104]. Gold is the most commonly used metal for bioimaging. The surface of AuNPs can be easily modified with various biomolecules such as peptides, proteins, antibodies, enzymes, and nucleic acids. These biomolecules can interact with specific cells or organelles in vivo, which makes it possible for AuNPs to be used for targeted optical imaging. Gao et al. reported a real-time in situ imaging of nucleus by AuNPs fabricated with bifunctional peptides constructed with both Au-binding affinity and nucleus-targeting ability. The bifunctional peptides showed strong binding affinity toward AuNPs and ensured good surface coverage of the nanoparticles, which made it stable and efficient for precise bioimaging of the nucleus in cells [105]. The Au-Se bond is considered as a better candidate than the Au–S bond to link the peptides and AuNPs due to the stronger ability against interference of intracellular thiol. Pan et al. prepared the Au-Se-peptide nanoprobes through a direct freezing process. The obtained nanoprobe was successfully applied to identify autophagy and apoptosis in chemotherapeutic drug treated cancer cells [106].

**Fig. 5:**
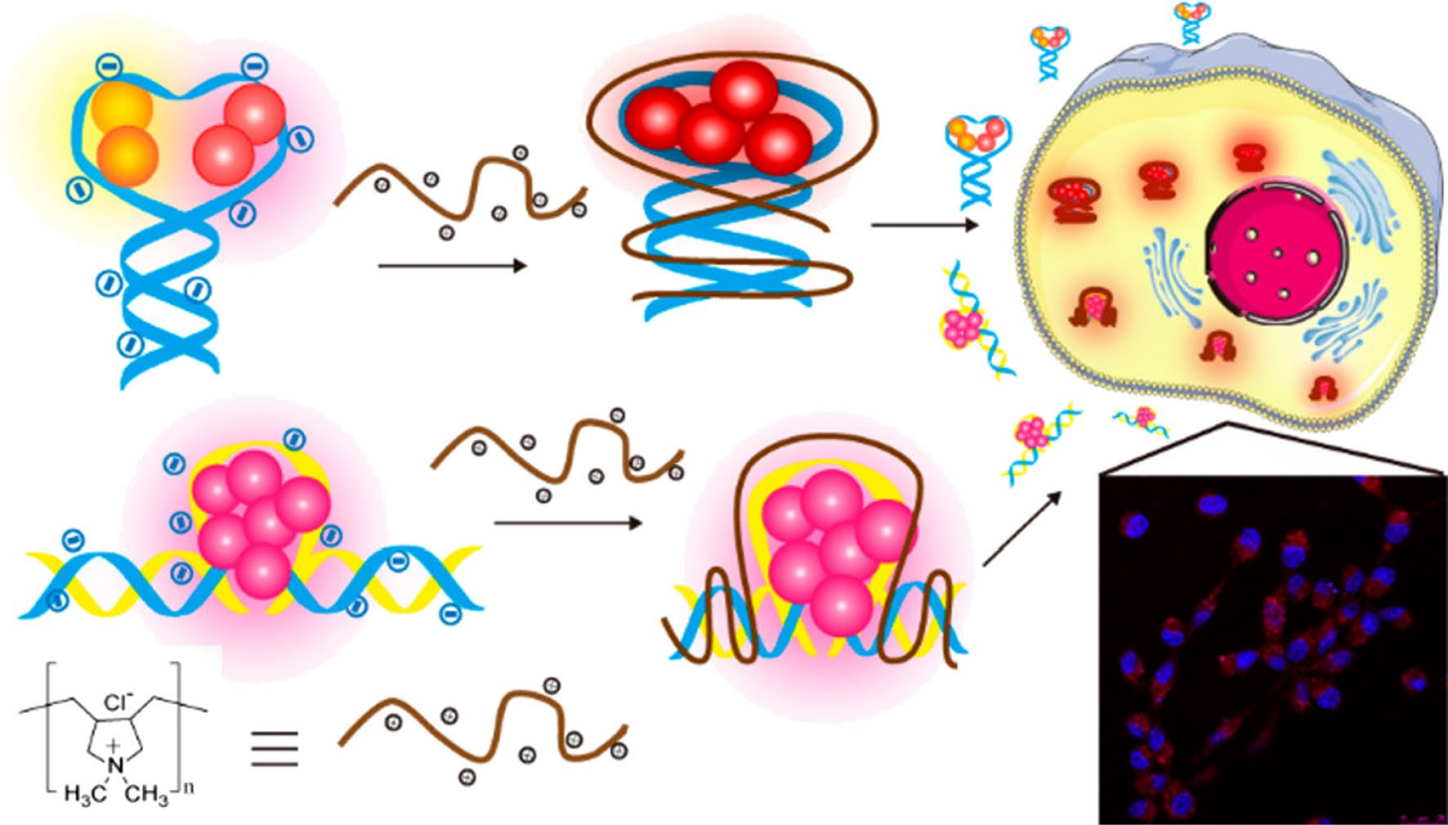
Formation of FL DNA–Ag NC–Cationic Polyelectrolyte Complexes for Cell Imaging [7]

As a novel fluorescent imaging technology, DNA-templated silver nanoclusters (DNA-Ag NCs) have aroused the attention of many scientists due to their unique properties, especially the tunable fluorescence emission range relying on DNA sequences. However, the highly negatively charged DNA backbones have always been a great obstacle for the expansive applications in bioimaging because of poor stability as well as poor cell permeability in physiological environment. It is also noteworthy that the PL property and fluorescent efficiency of DNA-Ag NCs are far from satisfying. As a result, figuring out how to neutralize the negative charge on the surface of DNA strands is of great urgency for researchers. Recently, Lyu and co-workers successfully modified fluorescent DNA-Ag NCs with cationic polyelectrolytes via electrostatic force between the positively charged polyelectrolytes and the negatively charged phosphate groups of the DNA strands, leading to a threefold fluorescence intensity enhancement [7] (Fig. 5). Li et al. reported a facile strategy to make gold nanoclusters with positive charge and silver nanoclusters with negative charge form aggregates by electrostatic interactions. An incredible 40-fold fluorescence intensity enhancement was obtained. Results demonstrated that the physiological stability improved a lot and the cell permeability was also enhanced, which promises its practical applications in the future.

#### Silica Nanoparticles for Bioimaging

Dye-doped fluorescent silica nanoparticles emerge with great potential for bioimaging as a novel and ideal platform for the monitoring of living cells and the whole body. The outer silica shell matrix protects fluorophores from outside chemical reaction factors as well as provides a hydrophilic shell for the inside insoluble nanoparticles, which renders the enhanced photo-stability and biocompatibility to the organic fluorescent dyes. Benefiting from the robust structure of silica matrices, dye-doped fluorescent silica nanoparticles have been presented with several superior properties including good biocompatibility, hydrophilic features, and high fluorescence intensity [107].

Jiao et al. also constructed a local hydrophobic cage in dye-doped fluorescent silica nanoparticles to improve their optical properties, which solves the problems of aggregation-caused quenching (ACQ) and poor photostability in aqueous media by organic fluorescence dyes benefiting from the robust structure of silica nanoparticles [108]. In addition, compared with free dyes, the fluorescent intensity both in water solution and living cells demonstrated a 12.3-fold enhancement due to the limitation of molecular motion, indicating a significant development for silica nanoparticles in biomedical applications. QDs have been developed for bioimaging both in vivo* and vitro* owing to their excellent optical qualities. However, a critical obstacle faced in QDs’ application in vivo is their poor biocompatibility. Inspired by the organic dye-conjugated silica-NPs, QDs-embedded silica-NPs have also been invented with the advantage that the excellent optical qualities of QDs can be retained, while the silica-NPs coat improves their biocompatibility to a large extent simultaneously. Darwish et al. reported that many QDs could be assembled around a central silica nanoparticle to form supra-NP assemblies. It was expected to be used for enhanced bioimaging because of their higher sensitivity and superior signal-to-background ratios [109]. There is reason to believe that silica-NPs conjugated with fluorescent nanomaterials with ideal optical properties will still be the dominant research interest in the future.

#### Phosphors for Bioimaging

For bioimaging, the sizes of phosphors need to be controlled so that they are small enough to be integrated with living cells. Furthermore, the aggregation of particles should be avoided for biocompatibility. Hence, the control of both particle sizes and dispersity in an aqueous solution is essential for the bioimaging application of the phosphors. Atabaev et al. prepared Eu, Gd-codoped Y_2_O_3_ phosphors which had a spherical morphology within the range 61–69 nm. Enhanced PL emission and low toxicity made these phosphors suitable for bioimgaing applications [110].

Upconversion nanomaterials are able to convert lower-energy near-infrared photons to higher-energy ones as emission. This anti-Stokes photoluminescence process will lead to low background noise, large tissue penetration depth, and low photo-damage in bioimaging applications [111]. Lanthanide-based phosphors are able to show upconversion emission owing to their photodurability and low phonon energy. Nallusamy et al. reported a NIR–NIR bioimaging system based on Er^3+^:Y_2_O_3_ phosphors by using NIR emission at 1550 nm under 980 nm excitation, which can allow a deeper penetration depth into biological tissues than ultraviolet or visible light excitation [112]. In addition, the surface of Er^3+^:Y_2_O_3_ was electrostatically PEGylated to improve the chemical durability and dispersion stability under physiological conditions. Thakur et al. synthesized Ho^3+^/Yb^3+^ co-doped GdVO_4_ phosphors via a modified sol–gel method. The prepared phosphors showed brilliant red upconversion emission under NIR excitation, which may be useful in bioimaging of the biomolecules [113].

#### Organic Frameworks for Bioimaging

Careful selection of MOF constituents can yield crystals of ultrahigh porosity and high thermal and chemical stability, with some of them being luminescent [114]. Recently, Sava Gallis’s group described a novel multifunctional MOF material platform which showed a wide spectral region from 614 to 1350 nm covering the deep red to NIR region. Both porosity and tunable emission properties made them highly suitable for in vivo bioimaging [115]. What’s more, to overcome the obstacle of MOF’s low selectivity towards malignant tissues, Liu et al. developed a target-induced bioimaging by conjugating DNA aptamers using ZrMOF nanoparticles as quenchers [116]. Based on the quenching of ZrMOF nanoparticles, target-induced bioimaging is achieved upon binding with the target.

### Biodetection

Since fluorescent nanomaterials can amplify the fluorescent signals significantly and be compatible with organisms, there is increasingly more research on their application in the rapid detection of biomolecules [117]. It will shorten the analysis time to a large extent if we are able to establish a real-time detection system by fluorescent nanomaterials. It has been discovered that multiple detection can be achieved by using QDs probes simultaneously [118, 119].

#### Pathogen Detection

Pathogens have been an unignorable threat to human health for centuries and these include many types of microorganisms ranging from bacteria (pathogenic *Escherichia coli, Salmonella, and Streptococcus pneumoniae*) and viruses (*Coronavirus, Influenza virus and hepatitis virus*). However, conventional methods for pathogen detection still need improvement of detection limits and detection speeds. For their applications in detecting pathogens, Tan and co-workers reported a bioconjugated nanoparticle-based biodetection for in situ pathogen quantification, which cost less than 20 min [120]. Tan’s success promises that quick and convenient pathogen detection is possible and can be achieved with these ingenious nanomaterials in the future. Here, we list diverse pathogens detected by fluorescent nanomaterials as shown in Table 2 [119, 121–129].

**Table 2:** Pathogen detection methods and properties of FNMs

Type of FNMs	Analytes	Mechanism	Detection limit	Ref
QDs	*Escherichia coli*	Antibody-conjugated	10^−3^ cfu/ml	[117]
	*Salmonella typhimurium*	Antibody-conjugated	10^−3^ cfu/ml	[117]
	*Shigella flexneri*	Antibody-conjugated	10^−3^ cfu/ml	[117]
	Respiratory Syncytial Virus	Protein labeling	35–50 pfu/ml	[118]
	*Bacillus thuringiensis Spores*	Analyte-conjugated	1,000 cfu/ml	[120]
	*Cryptosporidium parvum*	Antibody-conjugated	N.A	[121]
	*Giardia lamblia*	Antibody-conjugated	N.A	[121]
	*V. parahaemolyticus*	Chromatophores labeling	7 cfu/ml	[122]
	*Pseudomonas aeruginosa*	FRET	100 cfu/ml	[123]
Silica NPs	*Mycobacterium tuberculosis*	Antibody-conjugated	3.5 × 10^3^ cells/ml	[119]
AUNPs	*Shigella spp.*	probe-modified	10^3^ cells/ml	[124]
AgNCs	*Escherichia coli*	DNA-modified	60 cfu/ml	[125]
MNPs	*Shigella spp.*	DNA-modified	10^3^ cells/ml	[124]
UCNPs	*E. coli ATCC 8739*	FERT	3 cfu/ml	[126]

#### Nucleic Acid Detection

**Fig. 6:**
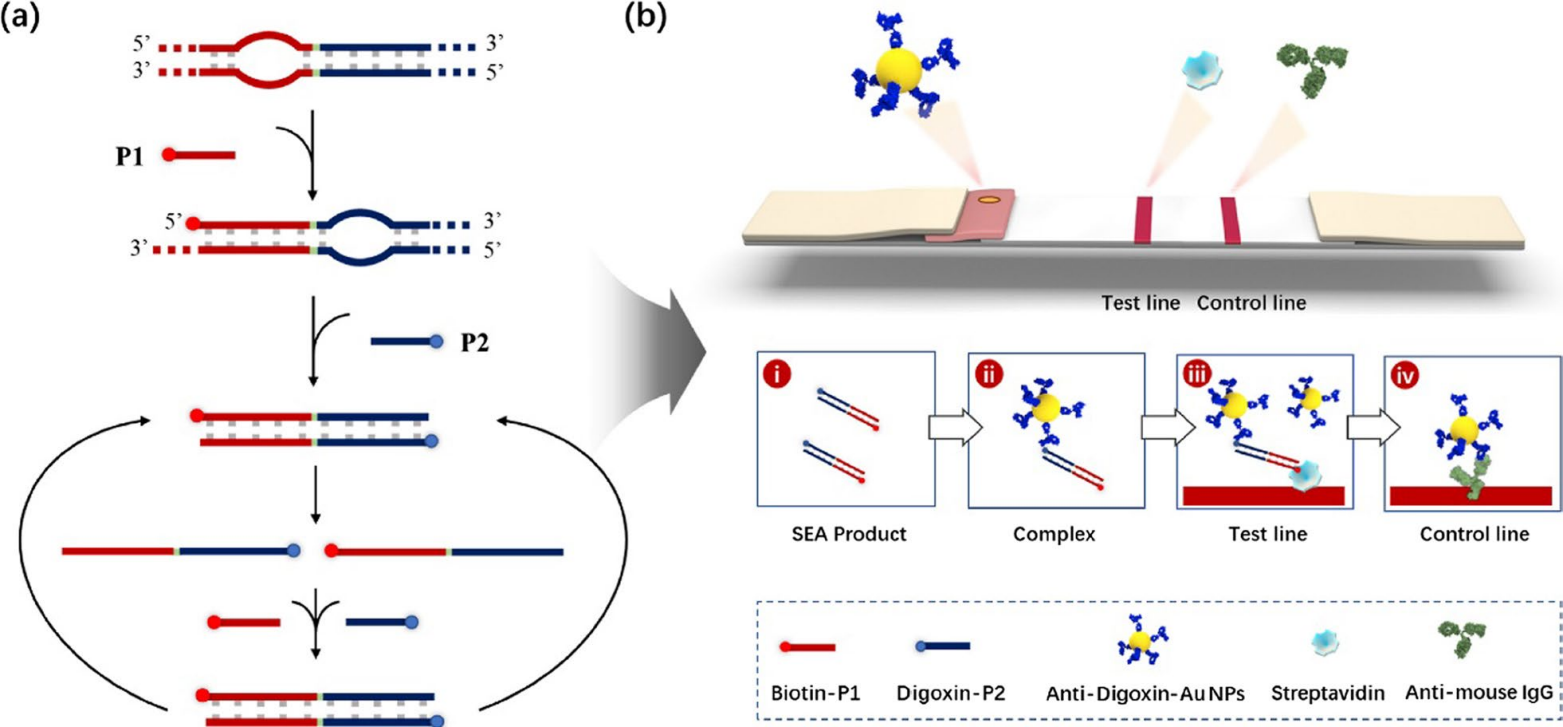
Schematic illustration of the SEA-LFA strip for the detection of nucleic acids [110]

Apart from pathogen detection, fluorescent nanomaterials have also aroused more and more interest of scientists in the detection of DNA. Owing to the merits that a number of biomolecules can be attached to the surface of fluorescent nanomaterials, the signal intensity of fluorescent nanomaterials in DNA detection can be enhanced significantly. Tan and co-workers developed an DNA detection method to detect gene products using bioconjugated dye-doped fluorescent silica nanoparticle with high sensitivity and photostability [130]. Although the analysis of nucleic acid has been successfully achieved by real-time nanomaterial fluorescence systems, there are still many shortcomings such as complex procedures or expensive instruments. To address these disadvantages, Wang’s group introduced a highly sensitive and visualized detection of nucleic acid by the combination of strand exchange amplification (SEA) and lateral flow assay strip (LFA) [131]. The system, which is possible to be widely used in areas requiring limited resource, is mainly characterized by integrating SEA with LFA (Fig. 6). There is no denying that the extremely high fluorescent signal for bioanalysis plays an irreplaceable role in these applications.

#### Drug Detection

In the field of drug analysis, a facile and low-cost analytical method is always in demand for high-speed detection of specific pharmaceutical compounds. Real-time detection of drugs can be achieved with selective and sensitive fluorescent nanomaterials owing to their outstanding optical properties. In the past decade, the modification of nanomaterials has lowered their detection limit and improved their detection accuracy significantly. Recently, it is reported that ampicillin can be detected in serum sample based on aptamer, its complementary strand (CS) and gold nanoparticles (AuNPs) [132]. The limit of detection (LOD) of this method can be as low as 29.2 pM. However, there are still many limitations in the detection of drugs or target molecules in vivo. Due to low selectivity, conventional fluorescent nanomaterials inevitably generate false positive results and adverse effects in vivo. In addition, current tracking systems can hardly realize real-time tracking because of insufficient labels and excitation sources. Considering the above limitations, a new method using up/down conversion (UC/DC) PL nanomaterials has attracted increasing attention. Seo et al. reported a single-photon-driven UC/DC system which demonstrated outstanding performance in the detection of heavy metal ion (i.e. Hg^2+^) in mussels [133]. LOD of the nanohybrids was *ca.* 1 nM. This system is appealing to researchers in the field of fluorescent nanomaterials for biomedical applications.

### Drug Delivery

**Fig. 7:**
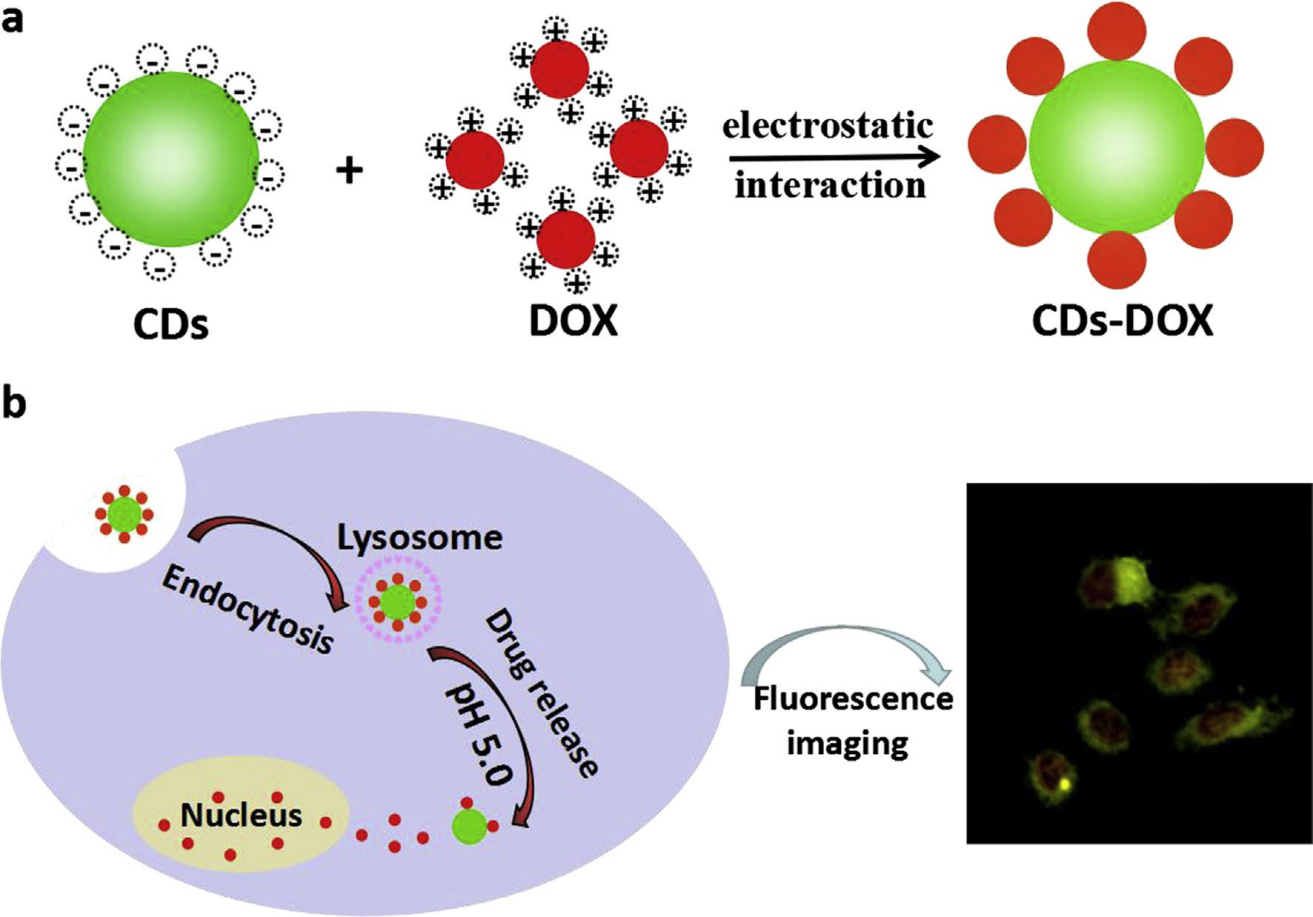
Schematic illustration of the preparation (**a**) and cellular uptake (**b**) of the CDs-DOX drug delivery system [113]

Until now, the technology of treating cancers with high efficiency and targeting function is not perfect enough. Under most circumstances, the anticancer drugs are distributed and released extensively in the body, which endangers the healthy cells and tissues irreversibly. Currently, a large variety of carriers for drug delivery have been designed. However, we can hardly supervise the distribution and result of the whole delivery process. Benefiting from the recent development of the surface modification technique, fluorescent materials capped with polymers like polyethylene glycol (PEG) can bond with drugs strongly and firmly. Then, the loaded drugs will be released in response to certain conditions such as pH, osmotic gradient and the surrounding environment. However, it should be confirmed whether the drugs are transported to the specific site or not. It’s also necessary for us to consider more details such as how much of the drugs is released in different positions. Aside from being drug carriers, fluorescent nanomaterials can also demonstrate the consequences of intracellular uptake due to their fluorescence property. QDs have been applied to monitor some important properties, such as delivery efficiency, release rate and distribution of drug molecules in vivo, which are beneficial for scientists in order to understand the specific targeting pathways of drug delivery within living cells. Duan and co-workers reported a facile pH-responsive fluorescent CDs drug delivery system [134]. Loaded with dox which is effective for gastric cancer, intracellular drug delivery and tracking could be simultaneously realized in patients (Fig. 7). The report highlighted the ability of fluorescent CDs to label and track the drug delivery process for at least 48 h, which showed a great potential in bioimaging, biolabeling and traceable drug delivery. Duan et al. designed a pH and receptor dual-responsive drug delivery system [135]. Hyaluronic acid was covalently attached to the surface of CDs, and doxorubicin was loaded by electrostatic self-assembly. In the tumor microenvironment (pH 5.6), the drug is released rapidly from the drug delivery system, while in the normal physiological environment (pH 7.4), the drug is hardly released. Endocytosis occurs when the drug delivery system reaches CD44 which is a receptor rich in tumor cells and can bind specifically to the hyaluronic acid. In addition, carbon nanotubes can be used for drug delivery by virtue of their high loading efficiency. Strong π-π interactions play a critical role in binding therapeutic agents with carbon nanotubes, which can be broke through changing external conditions, resulting in the release of drugs in specific position. Pennetta et al. functionalized single and multi-walled carbon nanotubes with a pyrrole derived compound to form a doxorubicin stacked drug delivery system. Biological studies showed that the synthesized nano-conveyors can effectively deliver the drug into cell lines and improve the therapeutic effects of doxorubicin [136].

### Photodynamic Therapy

**Fig. 8:**
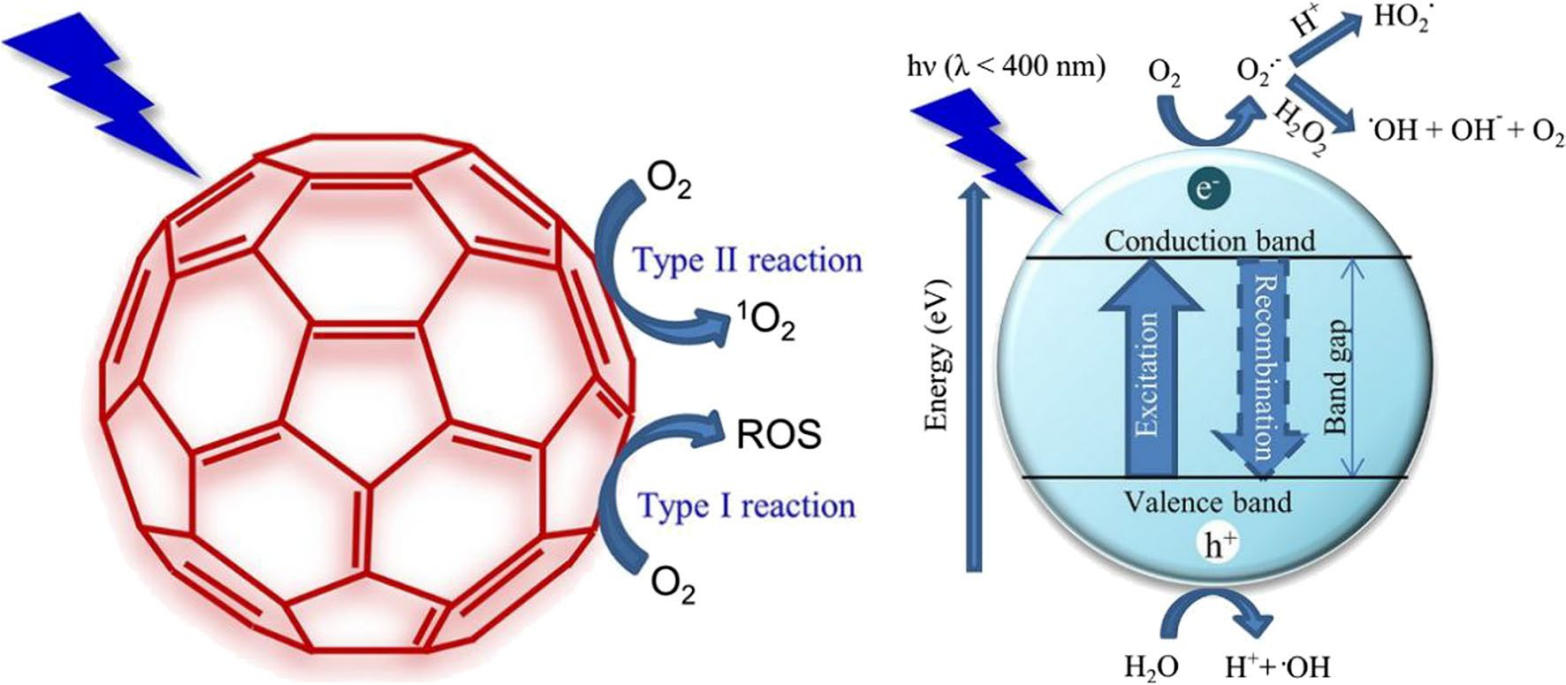
Schematic illustration of producing reactive oxygen species (ROS) for the photodynamic therapy (PDT) [119]

Photodynamic therapy (PDT) is a novel therapy method for tumors which utilizes the interaction between light and photosensitizer. In PDT, reactive oxygen species (ROS) is produced from oxygen by photosensitizers in the condition of specific wavelengths of light (mostly in the area of near infrared light). The specific mechanism is presented in Fig. 8. ROS includes singlet oxygen, superoxide radicals, hydrogen peroxide, and hydroxyl radicals that possess strong cytotoxicity which cause significant destruction of tumor cells. However, there exist many defects such as limited penetration depth [137], hydrophobic properties [138], photobleaching [139], complicated procedure [140], and tumor hypoxia. PDT agents can hardly be dissolved and they will disperse extensively in vivo once they are taken, making it impossible to be targeted and selected. Fluorescent nanomaterial based photodynamic therapy developed fast in recent years [141]. Combined with the unique properties that QDs possess, such as high fluorescent efficiency and great spectral resolution, the effect of PDT can be enhanced. Barberi-Heyob, M and coworkers significantly enhanced the photodynamic efficiency with a concentration of 8 nM because of the light dose-dependent response [142]. In addition, photodynamic therapy can sometimes do harm to the skin and eyes of patients due to its photosensitive side-effect. To alleviate these adverse effects, a novel nanoparticle-based drug carrier for photodynamic therapy is reported which can provide stable aqueous dispersion of hydrophobic photosensitizers. Meanwhile, the key step of photogeneration of singlet oxygen was preserved, which is necessary for photodynamic action [143]. It is obvious that QDs combined photodynamic therapy will replace the conventional PDT someday.

## Challenges

### Synthesis Challenges

#### Achieving Uniform Distribution

In the synthesis process, the diameter and size distributions of FNMs can be hardly distributed uniformly due to the agglomeration of small particles. This could be fatal to the optical properties of FNMs in biomedical application. For this reason, the applications of FNMs are still at the laboratory scale. It has been confirmed that the surface properties primarily determine the agglomeration state of the nanoparticles and their size. Therefore, surface modification is promising to achieve uniform distribution of FNMs by altering their surface properties [144]. To date, silanized QDs have been widely used because the polymerized silica coating increases the stability in buffers under physiological conditions [145]. Carbon dots synthesized by hydrothermal reaction using water-soluble base were reported to be difficult to control the size and distribution of grain boundary [146]. Khanam et al. reported a facile and novel synthetic method for the preparation of hydroxyl capped CDs using an organic base and a surfactant (Triton X-100) to modify the surface. A narrow particle size distribution at 7.2 nm was found in Raman and DLS studies, which is smaller than the majority of the particles falling within the range of below 10 nm in diameter [147].

#### Fluorescence Quantum Yield

Fluorescence quantum yield plays a crucial role for FNMs in their efficiency for on-demand light emission. Tunable and highly fluorescent CDs can be prepared with the surface functionalization approach. Nitrogen-doped FNMs are reported to have improved fluorescence quantum yield. With increasing nitrogen content, fluorescence quantum yield can be increased to as high as 56% at high synthesis temperature [148]. A facile strategy was also developed to tune the photoluminescent properties of CDs using a microwave irradiation, with citric acid and nitrogen-containing branched polyethyleneimine (b-PEI) as precursors. At intermediate levels of b-PEI, the CDs produced a high photoluminescence yield [149]. Lin et al. explored carbon dots with a high-fluorescence quantum yield rate synthesized from L-cysteine and citric acid by the microwave-assisted method. The obtained carbon dots exhibited a high-fluorescence quantum yield (up to 85%), which is due to the combination of amidogens and sulfydryl with carbon dots, and henceforth bringing the improved fluorescence property [150]. The above examples demonstrate that nitrogen or other electron-rich atoms like sulphur can obtain satisfying fluorescence quantum yield.

#### Aggregation-Caused Quenching

Fluorescent molecules can emit light with high efficiency in dilute solution. However, in concentrated solution or solid state, their fluorescence will be weakened or even disappear. This phenomenon is called Aggregation-Caused Quenching (ACQ) [151]. This problem has been puzzling scientists for almost 150 years, thus hindered the extensive application of fluorescent dyes.

In order to make effective use of fluorescent dyes, scientists have attempted many methods. Most of them focused on reducing the concentration of fluorescent dyes to prevent ACQ effect. Tang et al. discovered the phenomenon of Aggregation-Induced Emission (AIE) [152]. Based on rationally designed molecules, the fluorescence of organic molecules in solid state can be attained. Still, for more than one hundred thousand different fluorescent dyes in the world, the problem of ACQ has not been completely resolved. As long as they aggregate together, ACQ will make them lose their fluorescent properties.

It is almost impossible for high concentration or solid state FNMs to show reliable fluorescence due to fluorescence quenching. Although fluorescence quenching can be used as a sensitive signal to indicate substrate concentration in analytical chemistry, [153] in the most circumstances, however, fluorescence quenching is undesirable for FNMs because it always has considerable influence on bioimaging and biodetection. To overcome this long-standing problem, Benson et al. reported a universal solution with the discovery of a class of materials called small-molecule ionic isolation lattices (SMILES) [154]. SMILES are simple to make by mixing cationic dyes with anion-binding cyanostar macrocycles. We draw inspiration from their findings and believe that similar results can be obtained if we replace cationic dyes with cationic modified FNMs.

### Application Challenges

#### Drawbacks of UV Light FNMs

Although FNMs realized the great-leap-forward from in vitro imaging to in vivo imaging, the emission fluorescence of most of FNMs is distributed in ultraviolet region or short wavelength visible region, which limits the optical imaging in living organisms. Moreover, use of UV light for monitoring living processes in cells and tissues has some potential drawbacks as long‐term irradiation of living cells may cause DNA damage and cell death. Therefore, the development of FNMs in near-infrared region is urgently needed in the future. Although NIR FNMs have deep tissue penetration, NIR detectors and filters are needed as the excitation and emission wavelengths are too close to each other, which restricts their range of application.

#### Interference in Biological Environment

Almost all biological tissues will produce significant autofluorescence under short wavelength, UV and visible light radiation [155]. Autofluorescence reduces the signal‐to‐background ratio and often interferes seriously with the visual effects. Some substances in the substrate of biological tissues also have great influence on the fluorescence, which reduces the selectivity of FNMs significantly. Until now, although the application of FNMs in mice showed acceptable outcomes, it is still difficult to achieve similar results in larger mammals. Much higher luminous efficiency under low power density excitation is required to avoid the background signal interference. Furthermore, temperature and pH conditions of the biological environments strongly affect the fluorescence of some substances as well. Therefore, satisfying fluorescence of FNMs at 37 °C and physiological pH should be guaranteed. It's worth noting that the pH in tumor is lower than normal tissues. Hence, fluorescence with high selectivity in acid environment will improve the efficiency of FNMs.

#### Biocompatibility

Biocompatibility refers to materials or systems that are nontoxic, safe and not causing physiological or immunological reactions. QDs with unique quantum confinement effect and electro-optical properties are attractive for biomedical applications. However, toxic effects of traditional semiconductor QDs made of heavy metal ions have serious safety concerns for their undesired environmental or health effects. In the purpose of circumventing this problem, core–shell structure modification of QDs by using biocompatible ligands or polymers is one way to effectively minimize toxic effects of traditional QDs. Furthermore, scientists are searching for heavy metal-free QDs formulations. Non-toxic or less toxic carbon dots and silica nanoparticles have shown their potential as the ideal FNMs for biomedical applications. Impurities brought in the process of syntheses may influence the biocompatibility of fluorescent nanomaterials. In order to reduce the influence of toxic impurities, green synthesis methods have been arousing the interest in biomedical fields. Chowdhury et al. utilized cacao extract which is a natural product as a reducing and stabilizing agent in the synthesis of gold nanoparticles [156]. Oxalic acid, as a constituent of cacao, can reduce Au^3+^ in HAuCl_4_ to metallic gold and stabilize the resultant nanoparticle colloidal solution. In vitro studies suggested that the cacao derived gold nanoparticles are biocompatible and suitable for biomedical applications. For MOFs, appropriate metal ions and ligands must be selected to lower the toxicity. Wang et al. employed Fe, Ti and Zr as constituents of MOFs, which are harmless and even beneficial elements to the body [157]. In vitro studies indicate that the proposed material has good biocompatibility and safety in biomedical application. What’s more, it is necessary to consider whether the difference in composition, surface charge, or modified group will have different biological effects. Taking these factors into account, we can improve the biocompatibility of FNMs with rational design.

## Conclusions

Benefiting from the unique properties of fluorescent nanomaterials, some limitations and barriers of conventional materials and methods in biomedical applications can be broken through. In this review, we comprehensively present the synthesis methods and applications of fluorescent nanomaterials. The advanced synthesis methods can offer us the fluorescent nanomaterials with ideal morphology, size ranges and structures. Meanwhile, the more convenient syntheses can lower the manufacturing cost of fluorescent nanomaterials, which is critical to their widespread applications in biomedical fields. Based on the improved synthesis techniques, the performance of fluorescent nanomaterials is bound to leap in their applications. With the development of fluorescent nanomaterials, bioimaging, biodection, drug delivery and photodynamic therapy will be more widely applied in the diagnosis and treatment of diseases. Finally, challenges in synthesis and biomedical applications point out exiting questions and developing direction. We hope that this review can bring some new insights to the development of fluorescent nanomaterials.

## Data Availability

All data and materials are available without restrictions.

## Abbreviations

FNMs: Fluorescent nanomaterials
PL: Photoluminescence
CDs: Carbon dots
QDs: Quantum dots
b-PEI: Branched polyethyleneimine
SWCNTs: Single-walled carbon nanotubes
CQDs: Carbon quantum dots
GQDs: Graphene quantum dots
NCQDs: Nitrogen-doped carbon quantum dots
NSCDs: Nitrogen and sulfur doped carbon dots
QY: Quantum yield
MCDs: Multi-doped carbon dots
MWCHTs: Multi-walled carbon nanotubes
GO: Graphene oxide
rGO: Reduced graphene oxide
CVD: Chemical vapor deposition
AuNPs: Gold nanoparticles
Ag NCs: Ag nanoclusters
Cu NCs: Cu nanoclusters
NPs: Nanoparticles
TEOS: Tetraethylorthosilicate
MR: Magnetic resonance
COFs: Covalent-organic frameworks
TNP: 2,4,6-Trinitrophenol
MOFs: Metal organic frameworks
ACQ: Aggregation-caused quenching
NIR: Near infrared
SEA: Strand exchange amplification
LFA: Lateral flow assay strip
CS: Complementary strand
LOD: Limit of detection
UC: Upconversion
DC: Downconversion
PDT: Photodynamic therapy
ROS: Reactive oxygen species
AIE: Aggregation-induced emission
SMILES: Small-molecule ionic isolation lattices

## References

[1] Soufi GJ, Iravani S (2020). Eco-friendly and sustainable synthesis of biocompatible nanomaterials for diagnostic imaging: current challenges and future perspectives. Green Chem.

[2] Koutsogiannis P, Thomou E, Stamatis H, Gournis D, Rudolf P (2020). Advances in fluorescent carbon dots for biomedical applications. Adv Phys-X.

[3] Kou XL, Jiang SC, Park SJ, Meng LY (2020). A review: recent advances in preparations and applications of heteroatom-doped carbon quantum dots. Dalton Trans.

[4] Zuo GC, Xie AM, Pan XH, Su T, Li JJ, Dong W (2018). Fluorine-doped cationic carbon dots for efficient gene delivery. ACS Appl Nano Mater.

[5] Gubala V, Giovannini G, Kunc F, Monopoli MP, Moore CJ (2020). Dye-doped silica nanoparticles: synthesis, surface chemistry and bioapplications. Cancer Nanotechnol.

[6] Pratiwi FW, Kuo CW, Chen BC, Chen PL (2019). Recent advances in the use of fluorescent nanoparticles for bioimaging. Nanomedicine.

[7] Lyu D, Li J, Wang XW, Guo WW, Wang EK (2019). Cationic-Polyelectrolyte-Modified Fluorescent DNA-Silver Nanoclusters with Enhanced Emission and Higher Stability for Rapid Bioimaging. Anal Chem.

[8] Jiang X, Röcker C, Hafner M, Brandholt S, Dörlich RM, Nienhaus GU (2010). Endo-and exocytosis of zwitterionic quantum dot nanoparticles by live HeLa cells. ACS Nano.

[9] Nozik AJ, Beard MC, Luther JM, Law M, Ellingson RJ, Johnson JC (2010). Semiconductor quantum dots and quantum dot arrays and applications of multiple exciton generation to third-generation photovoltaic solar cells. Chem Rev.

[10] Efros AL, Efros AL (1982). Interband absorption of light in a semiconductor sphere. Soviet Phys Semicond USSR.

[11] Ekimov A, Onushchenko A (1982). Quantum size effect in the optical-spectra of semiconductor micro-crystals. Soviet Phys Semicond USSR.

[12] Bajorowicz B, Kobylanski MP, Golabiewska A, Nadolna J, Zaleska-Medynska A, Malankowska A (2018). Quantum dot-decorated semiconductor micro- and nanoparticles: a review of their synthesis, characterization and application in photocatalysis. Adv Colloid Interface Sci.

[13] Dabbousi B, Rodriguez-Viejo J, Mikulec FV, Heine J, Mattoussi H, Ober R, Jensen K, Bawendi M (1997). (CdSe) ZnS core-shell quantum dots: synthesis and characterization of a size series of highly luminescent nanocrystallites. J Phys Chem B.

[14] Gerion D, Pinaud F, Williams SC, Parak WJ, Zanchet D, Weiss S, Alivisatos AP (2001). Synthesis and properties of biocompatible water-soluble silica-coated CdSe/ZnS semiconductor quantum dots. J Phys Chem B.

[15] Guo WH, Li JJ, Wang YA, Peng XG (2003). Luminescent CdSe/CdS core/shell nanocrystals in dendron boxes: superior chemical, photochemical and thermal stability. J Am Chem Soc.

[16] Yang YK, Chang YY, Guo YY, Yu LG, Zhang GH, Zhai DD, Wang XM, Sun XT (2019). Fluorometric microplate-based dimethoate assay using CdSe/ZnS quantum dots coated with a molecularly imprinted polymer. Microchim Acta.

[17] Hines MA, Guyot-Sionnest P (1996). Synthesis and characterization of strongly luminescing ZnS-capped CdSe nanocrystals. J Phys Chem.

[18] Xu X, Ray R, Gu Y, Ploehn HJ, Gearheart L, Raker K, Scrivens WA (2004). Electrophoretic analysis and purification of fluorescent single-walled carbon nanotube fragments. J Am Chem Soc.

[19] Li SQ, Pan RF, Mehdi YA, Xiao DL, He H (2015). One-step spontaneous synthesis of fluorescent carbon nanoparticles with thermosensitivity from polyethylene glycol. New J Chem.

[20] Zuo PL, Lu XH, Sun ZG, Guo YH, He H (2016). A review on syntheses, properties, characterization and bioanalytical applications of fluorescent carbon dots. Microchim Acta.

[21] Baker SN, Baker GA (2010). Luminescent carbon nanodots: emergent nanolights. Angewandte Chemie-Int Ed.

[22] Zhao QL, Zhang ZL, Huang BH, Peng J, Zhang M, Pang DW (2008). Facile preparation of low cytotoxicity fluorescent carbon nanocrystals by electrooxidation of graphite. Chem Commun.

[23] Li Y, Hu Y, Zhao Y, Shi GQ, Deng LE, Hou YB, Qu LT (2011). An electrochemical avenue to green-luminescent graphene quantum dots as potential electron-acceptors for photovoltaics. Adv Mater.

[24] Liu HP, Ye T, Mao CD (2007). Fluorescent carbon nanoparticles derived from candle soot. Angewandte Chemie-Int Ed.

[25] Tao HQ, Yang K, Ma Z, Wan JM, Zhang YJ, Kang ZH, Liu Z (2012). In vivo NIR fluorescence imaging, biodistribution, and toxicology of photoluminescent carbon dots produced from carbon nanotubes and graphite. Small.

[26] Yang Y, Cui J, Zheng M, Hu C, Tan S, Xiao Y, Yang Q, Liu Y (2012). One-step synthesis of amino-functionalized fluorescent carbon nanoparticles by hydrothermal carbonization of chitosan. Chem Commun (Camb).

[27] Xiao DL, Yuan DH, He H, Gao MM (2013). Microwave assisted one-step green synthesis of fluorescent carbon nanoparticles from ionic liquids and their application as novel fluorescence probe for quercetin determination. J Lumines.

[28] Tang LB, Ji RB, Cao XK, Lin JY, Jiang HX, Li XM, Teng KS, Luk CM, Zeng SJ, Hao JH, Lau SP (2012). Deep ultraviolet photoluminescence of water-soluble self-passivated graphene quantum dots. ACS Nano.

[29] da Silva J, Goncalves HMR (2011). Analytical and bioanalytical applications of carbon dots. Trac-Trends Anal Chem.

[30] Yan X, Cui X, Li L-s (2010). Synthesis of large, stable colloidal graphene quantum dots with tunable size. J Am Chem Soc.

[31] Zong J, Zhu Y, Yang X, Shen J, Li C (2011). Synthesis of photoluminescent carbogenic dots using mesoporous silica spheres as nanoreactors. Chem Commun.

[32] Simões EFC, Leitão JMM, da Silva JCGE (2016). Carbon dots prepared from citric acid and urea as fluorescent probes for hypochlorite and peroxynitrite. Microchim Acta.

[33] Xiao D, Yuan D, He H, Gao M (2013). Microwave assisted one-step green synthesis of fluorescent carbon nanoparticles from ionic liquids and their application as novel fluorescence probe for quercetin determination. J Lumin.

[34] Xu H, Zhang K, Liu Q, Liu Y, Xie M (2017). Visual and fluorescent detection of mercury ions by using a dually emissive ratiometric nanohybrid containing carbon dots and CdTe quantum dots. Microchim Acta.

[35] Esteves da Silva JCG, Goncalves HMR (2011). Analytical and bioanalytical applications of carbon dots. Trac-Trends Anal Chem.

[36] Romero V, Vila V, de la Calle I, Lavilla I, Bendicho C (2019). Turn–on fluorescent sensor for the detection of periodate anion following photochemical synthesis of nitrogen and sulphur co–doped carbon dots from vegetables. Sens Actuators B Chem.

[37] Feng XT, Ashley J, Zhou TC, Sun Y (2018). Fluorometric determination of doxycycline based on the use of carbon quantum dots incorporated into a molecularly imprinted polymer. Microchim Acta.

[38] Pathak A, Suneesh PV, Stanley J, Babu TGS (2019). Multicolor emitting N/S-doped carbon dots as a fluorescent probe for imaging pathogenic bacteria and human buccal epithelial cells. Microchim Acta.

[39] Parvin N, Mandal TK (2017). Dually emissive P, N-co-doped carbon dots for fluorescent and photoacoustic tissue imaging in living mice. Microchim Acta.

[40] Zhang Y, Chan KF, Wang B, Chiu PWY, Zhang L (2018). Spore-derived color-tunable multi-doped carbon nanodots as sensitive nanosensors and intracellular imaging agents. Sens Actuators B Chem.

[41] Yan FY, Bai ZJ, Zu FL, Zhang Y, Sun XD, Ma TC, Chen L (2019). Yellow-emissive carbon dots with a large Stokes shift are viable fluorescent probes for detection and cellular imaging ofsilver ions and glutathione. Microchim Acta.

[42] Butt MTZ, Preuss K, Titirici MM, Rehman HU, Briscoe J (2018). Biomass-derived nitrogen-doped carbon aerogel counter electrodes for dye sensitized solar cells. Materials.

[43] Liu GX, Li BQ, Liu Y, Feng YJ, Jia DC, Zhou Y (2019). Rapid and high yield synthesis of carbon dots with chelating ability derived from acrylamide/chitosan for selective detection of ferrous ions. Appl Surf Sci.

[44] Li X, Wang H, Shimizu Y, Pyatenko A, Kawaguchi K, Koshizaki N (2011). Preparation of carbon quantum dots with tunable photoluminescence by rapid laser passivation in ordinary organic solvents. Chem Commun.

[45] Gokus T, Nair RR, Bonetti A, Boehmler M, Lombardo A, Novoselov KS, Geim AK, Ferrari AC, Hartschuh A (2009). Making Graphene Luminescent by Oxygen Plasma Treatment. ACS Nano.

[46] Molkenova A, Toleshova A, Song SJ, Kang MS, Abduraimova A, Han DW, Atabaev TS (2020). Rapid synthesis of nontoxic and photostable carbon nanoparticles for bioimaging applications. Mater Lett.

[47] Roy S, Korzeniowska B, Dixit CK, Manickam G, Daniels S, McDonagh C (2015). Biocompatibility and bioimaging application of carbon nanoparticles synthesized by phosphorus pentoxide combustion method. J Nanomater.

[48] Ghosh S, Ali H, Jana NR (2019). Water dispersible red fluorescent carbon nanoparticles via carbonization of resorcinol. ACS Sustain Chem Eng.

[49] Prajapati SK, Malaiya A, Kesharwani P, Soni D, Jain A. Biomedical applications and toxicities of carbon nanotubes. Drug Chem Toxicol. 10.1080/01480545.2019.1709492 10.1080/01480545.2019.1709492 31908176

[50] Karimi P (2016). Effects of structure and partially localization of the pi electron clouds of single-walled carbon nanotubes on the cation-pi Interactions. Iran J Chem Chem Eng Int English Ed.

[51] Nagai Y, Yudasaka M, Kataura H, Fujigaya T (2019). Brighter near-IR emission of single-walled carbon nanotubes modified with a cross-linked polymer coating. Chem Commun.

[52] Roslan MS, Chaudhary KT, Doylend N, Agam A, Kamarulzaman R, Haider Z, Mazalan E, Ali J (2019). Growth of wall-controlled MWCNTs by magnetic field assisted arc discharge plasma. J Saudi Chem Soc.

[53] Mwafy EA, Mostafa AM (2019). Multi walled carbon nanotube decorated cadmium oxide nanoparticles via pulsed laser ablation in liquid media. Opt Laser Technol.

[54] Zhao H, Tu N, Zhang WB, Zhang M, Wang J (2021). Novel synthesis of Silicon/Carbon nanotubes microspheres as anode additives through chemical vapor deposition in fluidized bed reactors. Scr Mater.

[55] Lee AJ, Wang XY, Carlson LJ, Smyder JA, Loesch B, Tu XM, Zheng M, Krauss TD (2011). Bright fluorescence from individual single-walled carbon nanotubes. Nano Lett.

[56] Hou Z, Krauss TD (2017). Photoluminescence brightening of isolated single-walled carbon nanotubes. J Phys Chem Lett.

[57] Sinclair RC, Suter JL, Coveney PV (2019). Micromechanical exfoliation of graphene on the atomistic scale. Phys Chem Chem Phys.

[58] Ma H, Shen ZG (2020). Exfoliation of graphene nanosheets in aqueous media. Ceram Int.

[59] Lee H, Choi JI, Park J, Jang SS, Lee SW (2020). Role of anions on electrochemical exfoliation of graphite into graphene in aqueous acids. Carbon.

[60] Liu ZZ, Xu QF, Zhang CTF, Sun QY, Wang CB, Dong MD, Wang ZG, Ohmori H, Kosinova M, Goto T, Tu R, Zhang S (2020). Laser-induced growth of large-area epitaxial graphene with low sheet resistance on 4H-SiC(0001). Appl Surf Sci.

[61] Wang JB, Ren Z, Hou Y, Yan XL, Liu PZ, Zhang H, Zhang HX, Guo JJ (2020). A review of graphene synthesis at low temperatures by CVD methods. New Carbon Mater.

[62] Lu ZC, Zhai XT, Yi RH, Li ZY, Zhang RX, Wei Q, Xing GC, Lu G, Huang W (2020). Photoluminescence emission during photoreduction of graphene oxide sheets as investigated with single-molecule microscopy. J Phys Chem C.

[63] Krishnamoorthy K, Veerapandian M, Mohan R, Kim SJ (2012). Investigation of Raman and photoluminescence studies of reduced graphene oxide sheets. Appl Phys a Mater Sci Process.

[64] Kuhn S, Hakanson U, Rogobete L, Sandoghdar V (2006). Enhancement of single-molecule fluorescence using a gold nanoparticle as an optical nanoantenna. Phys Rev Lett.

[65] Sonnichsen C, Reinhard BM, Liphardt J, Alivisatos AP (2005). A molecular ruler based on plasmon coupling of single gold and silver nanoparticles. Nat Biotechnol.

[66] Karimadom BR, Kornweitz H (2021). Mechanism of producing metallic nanoparticles, with an emphasis on silver and gold nanoparticles, using bottom-up methods. Molecules.

[67] Zhang J, Li C, Zhang X, Huo S, Jin S, An F-F, Wang X, Xue X, Okeke CI, Duan G, Guo F, Zhang X, Hao J, Wang PC, Zhang J, Liang X-J (2015). In vivo tumor-targeted dual-modal fluorescence/CT imaging using a nanoprobe co-loaded with an aggregation-induced emission dye and gold nanoparticles. Biomaterials.

[68] Chansuvarn W, Tuntulani T, Imyim A (2015). Colorimetric detection of mercury(II) based on gold nanoparticles, fluorescent gold nanoclusters and other gold-based nanomaterials. Trac-Trends Anal Chem.

[69] Li J, Zhu J-J, Xu K (2014). Fluorescent metal nanoclusters: From synthesis to applications. TrAC Trends Anal Chem.

[70] Kawasaki H, Kosaka Y, Myoujin Y, Narushima T, Yonezawa T, Arakawa R (2011). Microwave-assisted polyol synthesis of copper nanocrystals without using additional protective agents. Chem Commun (Camb).

[71] Rotaru A, Dutta S, Jentzsch E, Gothelf K, Mokhir A (2010). Selective dsDNA-templated formation of copper nanoparticles in solution. Angew Chem Int Ed Engl.

[72] Aparna RS, Anjali Devi JS, Anjana RR, Nebu J, George S (2019). Zn(II) ion modulated red emitting copper nanocluster probe for the fluorescence turn on sensing of RDX. Sensors Actuators B Chem.

[73] Tummala S, Huang WA, Wu BH, Chang KC, Ho YP (2020). Fluorescent mesoporous nanoparticles for beta-lactamase screening assays. Chemistryopen.

[74] Stöber W, Fink A, Bohn E (1968). Controlled growth of monodisperse silica spheres in the micron size range. J Colloid Interface Sci.

[75] Finnie KS, Bartlett JR, Barbé CJA, Kong L (2007). Formation of silica nanoparticles in microemulsions. Langmuir.

[76] He C, Zhu W, Xu Y, Zhong Y, Zhou J, Qian X (2010). Ratiometric and reusable fluorescent nanoparticles for Zn2+ and H 2 PO 4− detection in aqueous solution and living cells. J Mater Chem.

[77] Wang Y, Gu HC (2015). Core-shell-type magnetic mesoporous silica nanocomposites for bioimaging and therapeutic agent delivery. Adv Mater.

[78] Gawande MB, Monga Y, Zboril R, Sharma RK (2015). Silica-decorated magnetic nanocomposites for catalytic applications. Coord Chem Rev.

[79] Liu JN, Bu WB, Shi JL (2015). Silica coated upconversion nanoparticles: a versatile platform for the development of efficient theranostics. Accounts Chem Res.

[80] Lee JE, Lee N, Kim T, Kim J, Hyeon T (2011). Multifunctional mesoporous silica nanocomposite nanoparticles for theranostic applications. Accounts Chem Res.

[81] Qin XY, Wang J, Yuan Q (2020). Synthesis and biomedical applications of lanthanides-doped persistent luminescence phosphors with NIR emissions. Front Chem.

[82] Yu MM, Chen XD, Mei GJ (2018). Hydrothermal synthesis and luminescent properties of Y2O3:Eu3+ from waste phosphors. Results Phys.

[83] Khan S, Choi Y, Ahn HY, Han JH, Ju BK, Chung J, Cho SH (2020). Control of particle size in flame spray pyrolysis of Tb-doped Y(2)O(3)for bio-imaging. Materials.

[84] Rocha LA, Campos-Junior PHA, Esbenshade J, Siqueira RL, Schiavon MA, Ferrari JL (2018). Biocompatibility and photoluminescence of Sm3+-doped SiO2-Gd2O3: a promising non-toxic red phosphor to plasmatic membrane tracking. Ceram Int.

[85] Perhaita I, Muresan LE, Tudoran LB, Silipas DT, Borodi G (2020). Synthesis of silicate apatite phosphors with enhanced luminescence via optimized precipitation technique through pH control. J Sol-Gel Sci Technol.

[86] Singh V, Tiwari MK (2020). UV emitting Pb2+ doped Ca2La8(SiO4)(6)O-2 phosphors prepared by sol-gel procedure. Optik.

[87] Wang J, Yan B (2019). Improving covalent organic frameworks fluorescence by triethylamine pinpoint surgery as selective biomarker sensor for diabetes mellitus diagnosis. Anal Chem.

[88] Yu L, Chen HX, Yue J, Chen XF, Sun MT, Hou J, Alamry KA, Marwani HM, Wang XK, Wang SH (2020). Europium metal-organic framework for selective and sensitive detection of doxycycline based on fluorescence enhancement. Talanta.

[89] Alivisatos P (2004). The use of nanocrystals in biological detection. Nat Biotechnol.

[90] Bruchez M, Moronne M, Gin P, Weiss S, Alivisatos AP (1998). Semiconductor nanocrystals as fluorescent biological labels. Science.

[91] Chen FQ, Gerion D (2004). Fluorescent CdSe/ZnS nanocrystal-peptide conjugates for long-term, nontoxic imaging and nuclear targeting in living cells. Nano Lett.

[92] Chan WCW, Nie SM (1998). Quantum dot bioconjugates for ultrasensitive nonisotopic detection. Science.

[93] Kameyama T, Yamauchi H, Yamamoto T, Mizumaki T, Yukawa H, Yamamoto M, Ikeda S, Uematsu T, Baba Y, Kuwabata S, Torimoto T (2020). Tailored Photoluminescence Properties of Ag(In, Ga)Se-2 Quantum Dots for Near-Infrared In Vivo Imaging. ACS Appl Nano Mater.

[94] de Arquer FPG, Talapin DV, Klimov VI, Arakawa Y, Bayer M, Sargent EH (2021). Semiconductor quantum dots: Technological progress and future challenges. Science.

[95] Gao WL, Song HH, Wang X, Liu X, Pang XB, Zhou Y, Gao B, Peng XJ (2018). Carbon dots with red emission for sensing of Pt2+, Au3+, and Pd2+ and their bioapplications in vitro and in vivo. ACS Appl Mater Interfaces.

[96] Li D, Jing PT, Sun LH, An Y, Shan XY, Lu XH, Zhou D, Han D, Shen DZ, Zhai YC, Qu SN, Zboril R, Rogach AL (2018). Near-infrared excitation/emission and multiphoton-induced fluorescence of carbon dots. Adv Mater.

[97] Yogesh GK, Shuaib EP, Roopmani P, Gumpu MB, Krishnan UM, Sastikumar D (2019). Fluorescent carbon nanoparticles from laser-ablated Bougainvillea alba flower extract for bioimaging applications. Appl Phys a-Mater Sci Process.

[98] Mandal AK, Wu XJ, Ferreira JS, Kim M, Powell LR, Kwon H, Groc L, Wang YH, Cognet L (2020). Fluorescent sp(3) defect-tailored carbon nanotubes enable NIR-II single particle imaging in live brain slices at ultra-low excitation doses. Sci Rep.

[99] Ceppi L, Bardhan NM, Na Y, Siegel A, Rajan N, Fruscio R, Del Carmen MG, Belcher AM, Birrer MJ (2019). Real-time single-walled carbon nanotube-based fluorescence imaging improves survival after debulking surgery in an ovarian cancer model. ACS Nano.

[100] Huth K, Glaeske M, Achazi K, Gordeev G, Kumar S, Arenal R, Sharma SK, Adeli M, Setaro A, Reich S, Haag R (2018). Fluorescent polymer-single-walled carbon nanotube complexes with charged and noncharged dendronized perylene bisimides for bioimaging studies. Small.

[101] Park S, Kim T, Jo D, Jung JS, Jo G, Park Y, Kang ES, Kim YH, Kim J, Kim K, Hyun H (2019). Bioengineered Short Carbon Nanotubes as Tumor-Targeted Carriers for Biomedical Imaging. Macromol Res.

[102] Sun P, Xu KB, Guang SY, Xu HY (2021). Monodisperse functionalized GO for high-performance sensing and bioimaging of Cu2+ through synergistic enhancement effect. Talanta.

[103] Potsi G, Bourlinos AB, Mouselimis V, Polakova K, Chalmpes N, Gournis D, Kalytchuk S, Tomanec O, Blonski P, Medved M, Lazar P, Otyepka M, Zboril R (2019). Intrinsic photoluminescence of amine-functionalized graphene derivatives for bioimaging applications. Appl Mater Today.

[104] Li JJ, Zhu JJ, Xu K (2014). Fluorescent metal nanoclusters: from synthesis to applications. Trac-Trends Anal Chem.

[105] Gao YY, Liu YL, Yan R, Zhou JF, Dong H, Hua X, Wang P (2020). Bifunctional peptide-conjugated gold nanoparticles for precise and efficient nucleus-targeting bioimaging in live cells. Anal Chem.

[106] Pan W, Liu XH, Wan XY, Li J, Li YH, Lu F, Li N, Tang B (2019). Rapid preparation of Au-Se-peptide nanoprobe based on a freezing method for bioimaging. Anal Chem.

[107] Niu DC, Li YS, Shi JL (2017). Silica/organosilica cross-linked block copolymer micelles: a versatile theranostic platform. Chem Soc Rev.

[108] Jiao L, Liu YZ, Zhang XY, Hong GB, Zheng J, Cui JN, Peng XJ, Song FL (2020). Constructing a Local Hydrophobic Cage in Dye-Doped Fluorescent Silica Nanoparticles to Enhance the Photophysical Properties. ACS Central Sci.

[109] Darwish GH, Asselin J, Tran MV, Gupta R, Kim H, Boudreau D, Algar WR (2020). Fully self-assembled silica nanoparticle-semiconductor quantum dot supra-nanoparticles and immunoconjugates for enhanced cellular imaging by microscopy and smartphone camera. ACS Appl Mater Interfaces.

[110] Atabaev TS, Lee JH, Shin YC, Han DW, Choo KS, Jeon UB, Hwang JY, Yeom JA, Kim HK, Hwang YH (2017). Eu, Gd-Codoped Yttria nanoprobes for optical and T-1-weighted magnetic resonance imaging. Nanomaterials.

[111] Liu Q, Feng W, Li FY (2014). Water-soluble lanthanide upconversion nanophosphors: Synthesis and bioimaging applications in vivo. Coord Chem Rev.

[112] Venkatachalam N, Yamano T, Hemmer E, Hyodo H, Kishimoto H, Soga K (2013). Er3+ -Doped Y2O3 nanophosphors for near-infrared fluorescence bioimaging applications. J Am Ceram Soc.

[113] Thakur H, Singh BP, Kumar R, Gathania AK, Singh SK, Singh RK (2020). Synthesis, structural analysis, upconversion luminescence and magnetic properties of Ho3+/Yb3+ co-doped GdVO4 nanophosphor. Mater Chem Phys.

[114] Furukawa H, Cordova KE, O'Keeffe M, Yaghi OM (2013). The chemistry and applications of metal-organic frameworks. Science.

[115] Sava Gallis DF, Rohwer LES, Rodriguez MA, Barnhart-Dailey MC, Butler KS, Luk TS, Timlin JA, Chapman KW (2017). Multifunctional, tunable metal-organic framework materials platform for bioimaging applications. ACS Appl Mater Interfaces.

[116] Liu Y, Hou WJ, Xia L, Cui C, Wan S, Jiang Y, Yang Y, Wu Q, Qiu LP, Tan WH (2018). ZrMOF nanoparticles as quenchers to conjugate DNA aptamers for target-induced bioimaging and photodynamic therapy. Chem Sci.

[117] Li XW, Chen LG (2016). Fluorescence probe based on an amino-functionalized fluorescent magnetic nanocomposite for detection of folic acid in serum. ACS Appl Mater Interfaces.

[118] Zhu H, Fan JL, Du JJ, Peng XJ (2016). Fluorescent probes for sensing and imaging within specific cellular organelles. Accounts Chem Res.

[119] Jin BR, Wang SR, Lin M, Jin Y, Zhang SJ, Cui XY, Gong Y, Li A, Xu F, Lu TJ (2017). Upconversion nanoparticles based FRET aptasensor for rapid and ultrasenstive bacteria detection. Biosens Bioelectron.

[120] Zhao XJ, Hilliard LR, Mechery SJ, Wang YP, Bagwe RP, Jin SG, Tan WH (2004). A rapid bioassay for single bacterial cell quantitation using bioconjugated nanoparticles. Proc Natl Acad Sci USA.

[121] Zhao Y, Ye MQ, Chao QG, Jia NQ, Ge Y, Shen HB (2009). Simultaneous detection of multifood-borne pathogenic bacteria based on functionalized quantum dots coupled with immunomagnetic separation in food samples. J Agric Food Chem.

[122] Bentzen EL, House F, Utley TJ, Crowe JE, Wright DW (2005). Progression of respiratory syncytial virus infection monitored by fluorescent quantum dot probes. Nano Lett.

[123] Duan N, Wu SJ, Wang J, Zou Y, Wang ZP (2019). Quantum dot-based F0F1-ATPase aptasensor for vibrio parahaemolyticus detection. Food Anal Meth.

[124] Qin DL, He XX, Wang KM, Tan WH (2008). Using fluorescent nanoparticles and SYBR Green I based two-color flow cytometry to determine Mycobacterium tuberculosis avoiding false positives. Biosens Bioelectron.

[125] Ikanovic M, Rudzinski WE, Bruno JG, Allman A, Carrillo MP, Dwarakanath S, Bhahdigadi S, Rao P, Kiel JL, Andrews CJ (2007). Fluorescence assay based on aptamer-quantum dot binding to Bacillus thuringiensis spores. J Fluoresc.

[126] Zhu L, Ang S, Liu WT (2004). Quantum dots as a novel immunofluorescent detection system for Cryptosporidium parvum and Giardia lamblia. Appl Environ Microbiol.

[127] Song MS, Sekhon SS, Shin WR, Kim HC, Min J, Ahn JY, Kim YH (2017). Detecting and discriminating shigella sonnei using an aptamer-based fluorescent biosensor platform. Molecules.

[128] Zheng LB, Qi P, Zhang D (2018). DNA-templated fluorescent silver nanoclusters for sensitive detection of pathogenic bacteria based on MNP-DNAzyme-AChE complex. Sens Actuator B-Chem.

[129] Gao R, Zhong ZT, Gao XM, Jia L (2018). Graphene oxide quantum dots assisted construction of fluorescent aptasensor for rapid detection of pseudomonas aeruginosa in food samples. J Agric Food Chem.

[130] Zhao XJ, Tapec-Dytioco R, Tan WH (2003). Ultrasensitive DNA detection using highly fluorescent bioconjugated nanoparticles. J Am Chem Soc.

[131] Wang XK, Wang XD, Shi C, Ma CP, Chen LX (2020). Highly sensitive visual detection of nucleic acid based on a universal strand exchange amplification coupled with lateral flow assay strip. Talanta.

[132] Esmaelpourfarkhani M, Abnous K, Taghdisi SM, Chamsaz M (2020). A novel turn-off fluorescent aptasensor for ampicillin detection based on perylenetetracarboxylic acid diimide and gold nanoparticles. Biosens Bioelectron.

[133] Seo SE, Park CS, Park SJ, Kim KH, Lee J, Kim J, Lee SH, Song HS, Ha TH, Kim JH, Yim HW, Kim HI, Kwon OS (2020). Single-photon-driven up-/down-conversion nanohybrids for in vivo mercury detection and real-time tracking. J Mater Chem A.

[134] Duan QQ, Ma Y, Che MX, Zhang BY, Zhang YX, Li Y, Zhang WD, Sang SB (2019). Fluorescent carbon dots as carriers for intracellular doxorubicin delivery and track. J Drug Deliv Sci Technol.

[135] Duan QQ, Ma L, Zhang BY, Zhang YX, Li XN, Wang T, Zhang WD, Li Y, Sang SB (2020). Construction and application of targeted drug delivery system based on hyaluronic acid and heparin functionalised carbon dots. Colloid Surf B-Biointerfaces.

[136] Pennetta C, Floresta G, Graziano ACE, Cardile V, Rubino L, Galimberti M, Rescifina A, Barbera V (2020). Functionalization of single and multi-walled carbon nanotubes with polypropylene glycol decorated pyrrole for the development of doxorubicin nano-conveyors for cancer drug delivery. Nanomaterials.

[137] Liang LU, Lu YQ, Zhang R, Care A, Ortega TA, Deyev SM, Qian Y, Zvyagin AV (2017). Deep-penetrating photodynamic therapy with KillerRed mediated by upconversion nanoparticles. Acta Biomater.

[138] Xu DD, You YQ, Zeng FY, Wang Y, Liang CY, Feng HH, Ma X (2018). Disassembly of hydrophobic photosensitizer by biodegradable zeolitic imidazolate framework-8 for photodynamic cancer therapy. ACS Appl Mater Interfaces.

[139] James NS, Cheruku RR, Missert JR, Sunar U, Pandey RK (2018). Measurement of cyanine dye photobleaching in photosensitizer cyanine dye conjugates could help in optimizing light dosimetry for improved photodynamic therapy of cancer. Molecules.

[140] Wu H, Minamide T, Yano T (2019). Role of photodynamic therapy in the treatment of esophageal cancer. Dig Endosc.

[141] Wang Y, Wu W, Mao D, Teh C, Wang B, Liu B (2020). Metal-organic framework assisted and tumor microenvironment modulated synergistic image-guided photo-chemo. Therapy.

[142] Morosini V, Bastogne T, Frochot C, Schneider R, Francois A, Guillemin F, Barberi-Heyob M (2011). Quantum dot-folic acid conjugates as potential photosensitizers in photodynamic therapy of cancer. Photochem Photobiol Sci.

[143] Roy I, Ohulchanskyy TY, Pudavar HE, Bergey EJ, Oseroff AR, Morgan J, Dougherty TJ, Prasad PN (2003). Ceramic-based nanoparticles entrapping water-insoluble photosensitizing anticancer drugs: A novel drug-carrier system for photodynamic therapy. J Am Chem Soc.

[144] Bantz C, Koshkina O, Lang T, Galla HJ, Kirkpatrick CJ, Stauber RH, Maskos M (2014). The surface properties of nanoparticles determine the agglomeration state and the size of the particles under physiological conditions. Beilstein J Nanotechnol.

[145] Mehta VN, Kailasa SK, Wu HF (2014). Surface modified quantum dots as fluorescent probes for biomolecule recognition. J Nanosci Nanotechnol.

[146] Liu LQ, Li YF, Zhan L, Liu Y, Huang CZ (2011). One-step synthesis of fluorescent hydroxyls-coated carbon dots with hydrothermal reaction and its application to optical sensing of metal ions. Sci China-Chem.

[147] Khanam A, Tripathi SK, Roy D, Nasim M (2013). A facile and novel synthetic method for the preparation of hydroxyl capped fluorescent carbon nanoparticles. Colloid Surf B-Biointerfaces.

[148] Kundu A, Lee J, Park B, Ray C, Sankar KV, Kim WS, Lee SH, Cho IJ, Jun SC (2018). Facile approach to synthesize highly fluorescent multicolor emissive carbon dots via surface functionalization for cellular imaging. J Colloid Interface Sci.

[149] Bhattacharyya S, Ehrat F, Urban P, Teves R, Wyrwich R, Doblinger M, Feldmann J, Urban AS, Stolarczyk JK (2017). Effect of nitrogen atom positioning on the trade-off between emissive and photocatalytic properties of carbon dots. Nat Commun.

[150] Lin HT, Huang J, Ding LY (2019). Preparation of carbon dots with high-fluorescence quantum yield and their application in dopamine fluorescence probe and cellular imaging. J Nanomater.

[151] Bünau GV, Birks JB (1970) Photophysics of aromatic molecules. Wiley-Interscience, London 1970. 704 Seiten. Preis: 210s, 74(12):1294–1295

[152] Luo JD, Xie ZL, Lam JWY, Cheng L, Chen HY, Qiu CF, Kwok HS, Zhan XW, Liu YQ, Zhu DB, Tang BZ (2001). Aggregation-induced emission of 1-methyl-1,2,3,4,5-pentaphenylsilole. Chem Commun.

[153] Li WJ, Jiang TT, Pu Y, Jiao XD, Tan WQ, Qin S (2019). Glucose biosensor using fluorescence quenching with chitosan-modified graphene oxide. Micro Nano Lett.

[154] Benson CR, Kacenauskaite L, VanDenburgh KL, Zhao W, Qiao B, Sadhukhan T, Pink M, Chen JS, Borgi S, Chen CH, Davis BJ, Simon YC, Raghavachari K, Laursen BW, Flood AH (2020). Plug-and-play optical materials from fluorescent dyes and macrocycles. Chem.

[155] Zipfel WR, Williams RM, Christie R, Nikitin AY, Hyman BT, Webb WW (2003). Live tissue intrinsic emission microscopy using multiphoton-excited native fluorescence and second harmonic generation. Proc Natl Acad Sci USA.

[156] Chowdhury NR, Cowin AJ, Zilm P, Vasilev K (2018). “Chocolate” gold nanoparticles-one pot synthesis and biocompatibility. Nanomaterials.

[157] Wang Q, Zhao Y, Shi Z, Sun X, Bu T, Zhang C, Mao Z, Li X, Wang L (2021). Chem Eng J.

